# Synthesis and Spectroscopic Analysis of Piperine- and Piperlongumine-Inspired Natural Product Scaffolds and Their Molecular Docking with IL-1β and NF-κB Proteins

**DOI:** 10.3390/molecules25122841

**Published:** 2020-06-19

**Authors:** Gabriel Zazeri, Ana Paula R. Povinelli, Cécile S. Le Duff, Bridget Tang, Marinonio L. Cornelio, Alan M. Jones

**Affiliations:** 1 Departamento de Física–IBILCE, Rua Cristovão Colombo, 2265 CEP 15054-000 São José do Rio Preto–São Paulo, Brazil; gabriel.zazeri@unesp.br (G.Z.); ana.povinelli@unesp.br (A.P.R.P.); 2 School of Pharmacy, University of Birmingham, Edgbaston B15 2TT, UK; 3 School of Chemistry, University of Birmingham, Edgbaston B15 2TT, UK; C.S.LeDuff@bham.ac.uk (C.S.L.D.); B.Tang@bham.ac.uk (B.T.)

**Keywords:** molecular docking, piperine, piperlongumine

## Abstract

Inspired by the remarkable bioactivities exhibited by the natural products, piperine and piperlongumine, we synthesised eight natural product-inspired analogues to further investigate their structures. For the first time, we confirmed the structure of the key cyclised dihydropyrazolecarbothioamide piperine analogues including the use of two-dimensional (2D) ^15^N-based spectroscopy nuclear magnetic resonance (NMR) spectroscopy. Prior investigations demonstrated promising results from these scaffolds for the inhibition of inflammatory response via downregulation of the IL-1β and NF-κB pathway. However, the molecular interaction of these molecules with their protein targets remains unknown. Ab initio calculations revealed the electronic density function map of the molecules, showing the effects of structural modification in the electronic structure. Finally, molecular interactions between the synthesized molecules and the proteins IL-1β and NF-κB were achieved. Docking results showed that all the analogues interact in the DNA binding site of NF-κB with higher affinity compared to the natural products and, with the exception of **9a** and **9b,** have higher affinity than the natural products for the binding site of IL-1β. Specificity for the molecular recognition of **3a**, **3c** and **9b** with IL-1β through cation–π interactions was determined. These results revealed **3a**, **3c**, **4a**, **4c** and **10** as the most promising molecules to be evaluated as IL-1β and NF-κB inhibitors.

## 1. Introduction

Nature is a creative machine for the design of bioactive molecules with the potential to become efficient drugs for the treatment of several diseases. In recent decades, such creative scaffolds have driven the design of new molecules with pharmacological potential, including the concept of diversity-oriented synthesis (DOS) [1,2]. In recent years, both piperine and piperlongumine have been explored because of their wide spectrum of biological activity [3,4,5,6,7,8].

Piperine is an alkaloid found in piper species such as *Piper nigrum* (black pepper) and *Piper longum* (long pepper). Piperine is not only used as a seasoning but also in various preparations of traditional medicine, including the oldest medical science, practiced in India since ancient times (Ayurveda) [9]. Piperine has been widely studied by the scientific community, because of its anti-inflammatory, anti-carcinogenic, immunomodulatory and hepatoprotective activities [3]. As an anti-inflammatory, piperine acts through the inhibition of IL-1β and NF-κB inflammation pathway, leading to the downregulation of pro-inflammatory proteins, such as iNOS and COX-2 [10,11,12].

Piperine-inspired molecules investigated in the present work have had their anti-inflammatory and anti-carcinogenic activity previously studied by Mathew et al. [13,14], with promising results. In particular, **4a** had a comparable anti-inflammatory effect to Diclofenac. Furthermore, **3c** and **4c** showed inhibitory effects on the HCT116 colon cancer cell line. However, their interactions with the IL-1β and NF-κB pathways have not been investigated.

Piperlongumine (PPL), is an alkaloid isolated from long pepper that is widely used in Indian traditional medicine [15]. The biological activities of PPL and its analogues include anti-inflammatory, anti-carcinogenic and anti-atherosclerotic activities, among others [6,7,8,16]. Similar to piperine, studies indicated that anti-inflammatory and anti-carcinogenic activities of PPL are a consequence of the inhibition of the NF-κB pathway [17,18,19,20].

PPL has been shown to bind to the unit p65 of NF-κB, inhibiting NF-κB/DNA binding activity and blocking the transcription of cytokines and other pro-inflammatory proteins [18].

Despite the investigations reported in the literature that show promising results of these natural-product-inspired analogues in the inhibition of inflammatory response via the downregulation of IL-1β and NF-κB pathway, the molecular interaction of these molecules with the protein targets are unknown.

Herein, we explored the binding environment of the synthesised analogues in NF-κB and IL-1β using molecular docking. For piperine-inspired molecules, we investigated the effect caused by the substitution of the piperidine for an aromatic ring in the piperine scaffold (**3a**), a nitroarene group (**3b**) and an aminoarene group (**3c**). We investigated the effect of the cyclisation of the piperine scaffold to form a dihydropyrazolecarbothioamide moiety (**4a** and **4c**). For PPL-inspired molecules, we investigated the effects of removing a Michael acceptor (**9a**), the conversion of a lactam to a ketone (**10**) and the influence of methoxy groups (**9b**).

We present an adapted synthesis of piperine- and PPL-inspired molecules including data from 2D ^1^H-^15^N HSQC and HMBC spectra, computational characterization of the electronic structure of the analogues using *ab initio* calculations and molecular docking of the confirmed structures with the proteins IL-1β and NF-κB.

## 2. Results and Discussion

### 2.1. Spectroscopic Characterization of Natural Product Inspired Analogues

**Scheme 1:**
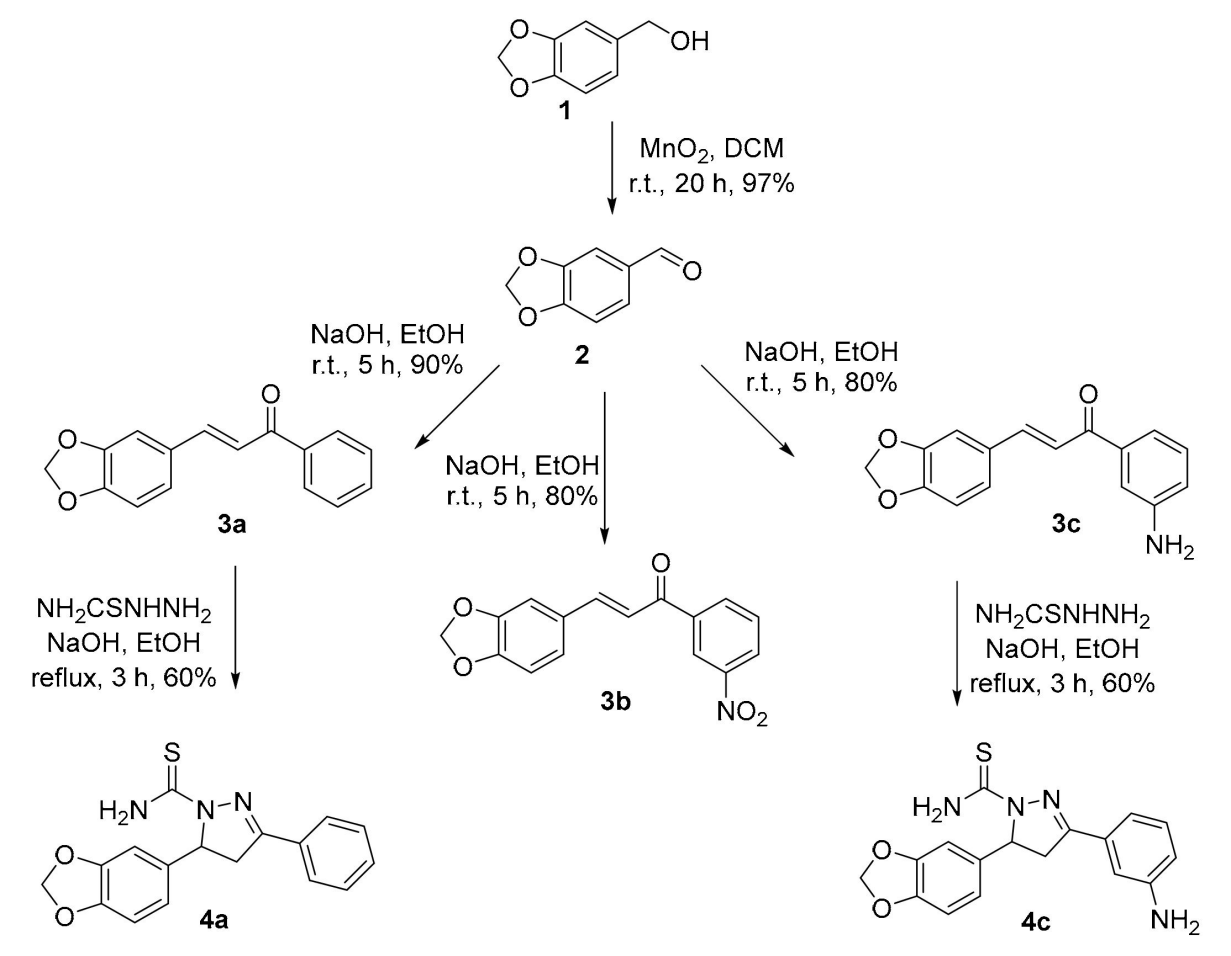
Synthesis of piperine-inspired molecules.

The synthesis of the piperine-inspired analogues began with a MnO_2_-mediated oxidation of piperonyl alcohol to the corresponding aldehyde with a 97% yield (Scheme 1). Claisen–Schmidt condensation of **2** with a variety of acetophenones afforded **3a**–**3c** in excellent yields (80–90%) after crystallisation. The initial synthetic route to **4c** required the synthesis of the nitro derivative (**3b**) followed by cyclisation with thiosemicarbazide and reduction, as the free amine may undergo unwanted side reactions during the cyclisation step. Unfortunately, all attempts to react **3b** with thiosemicarbazide resulted in recovered starting material. However, for the parent unsubstituted compound (**3a**) a cyclised compound (**4a**) was formed with 60% yield. Pleasingly, the unprotected amine containing molecule **3c** was smoothly converted to the cyclised molecule **4c** with 60% yield, mitigating the need to pursue **3b** further.

**Scheme 2:**
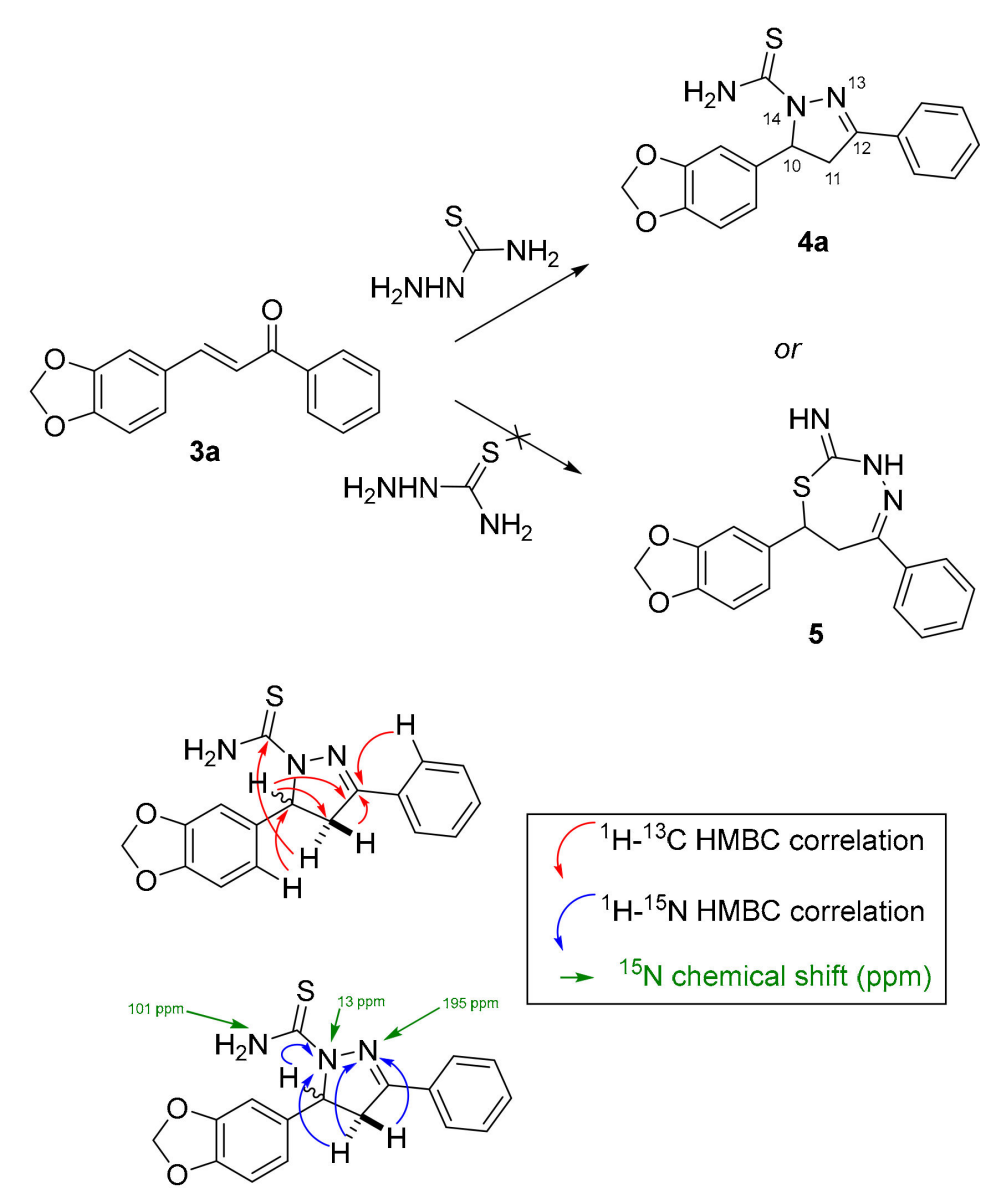
Two potential cyclisation outcomes upon **3a** and the selected instructive NMR correlations for **4a** in the 2D ^1^H-^13^C HMBC and 2D ^1^H-^15^N HMBC spectra.

The syntheses of piperine analogues have been reported by Mathew et al. [13] but with limited spectroscopic characterization (^1^H NMR and IR spectroscopy) of molecules of type **4**. We therefore performed re-synthesis prior to the computational study to confirm the connectivity of the new ring system. There is a potential for either a five-membered product (**4a**), or a seven-membered ring system (**5**) to form (Scheme 2). Selected instructive 2D ^1^H-^13^C HMBC and ^1^H-^15^N HMBC NMR spectra connectivities are shown in Scheme 2, along with the ^15^N NMR chemical shifts. Critically, the results showed the correct connectivity for a five-membered dihydropyrazolecarbothioamide system product (**4a** not **5**). **4a** was found to slowly degrade upon standing in CDCl_3_ at room temperature for an extended period of time.

**Scheme 3:**
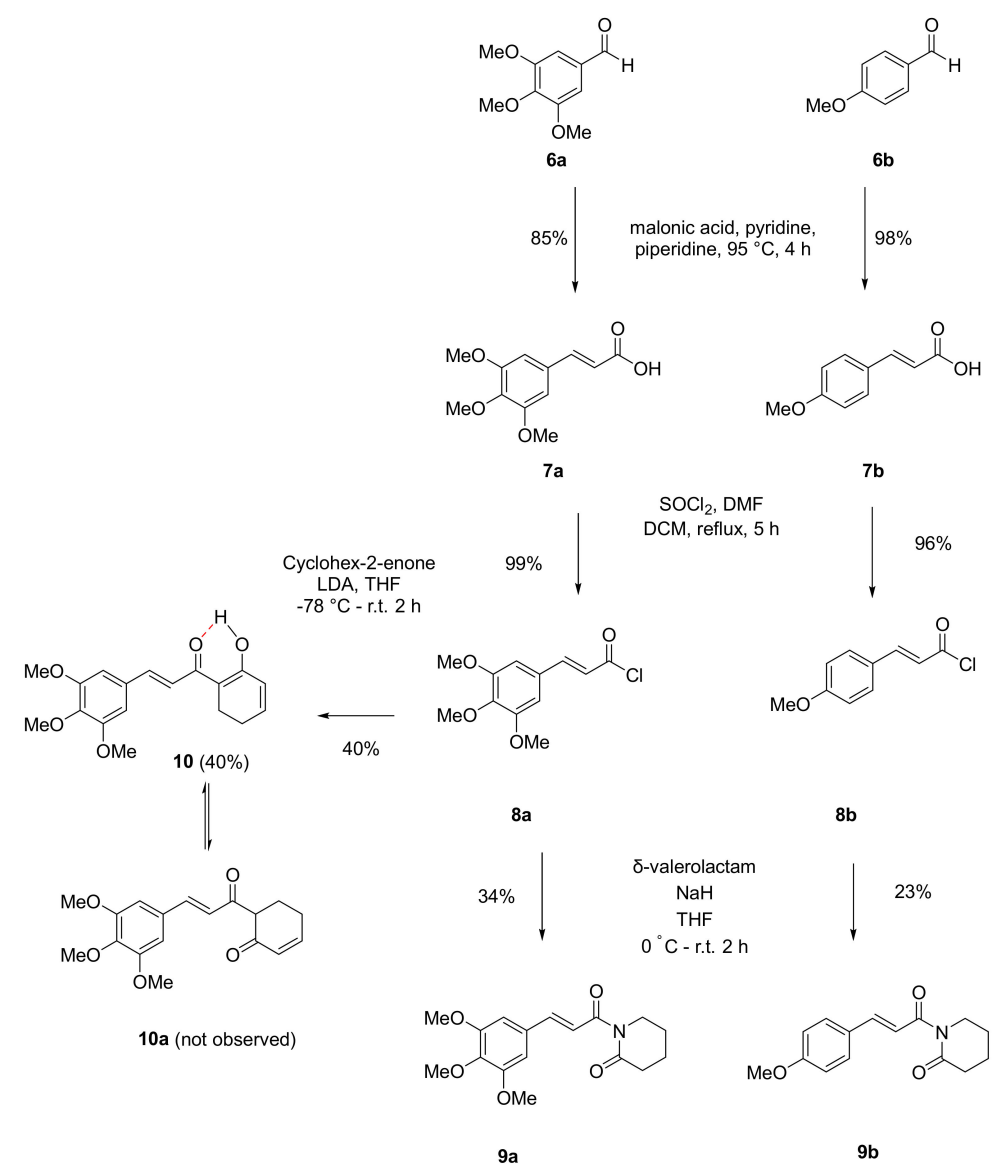
Synthesis of piperlongumine-inspired molecules.

The synthesis of the PPL-inspired analogues (Scheme 3) used a one-pot Doebner-modified Knoevenagel condensation and concomitant decarboxylation, to prepare the key acrylic acids (**7a,b**) from the appropriate benzaldehydes. Reaction of the acrylic acids with thionyl chloride under Vilsmeier–Haack conditions gave the short-lived vinylogous acyl chlorides (**8a,b**) which were reacted with either sodium 2-oxopiperidin-1-ide (derived from δ-valerolactam) or with lithium cyclohex-1-en-1-olate (derived from cyclohexanone), to afford the final products **9a**,**b** and **10** with modest isolated yields.

Although **10a** has been reported in the literature, we identified a previously unobserved ^1^H NMR spectroscopic signal at 15.76 ppm (Supplementary Materials), indicative of an intramolecular hydrogen bond occurring through tautomerization to give the stabilized structure, **10**.

### 2.2. Electronic Structures of Piperine and PPL-Inspired Molecules

*Ab initio* calculations were performed to obtain the structural parameterisation to be used in the molecular docking calculations. In addition, these calculations provided information about the electronic structure that contributed to the characterisation of the compounds.

**Figure 1:**
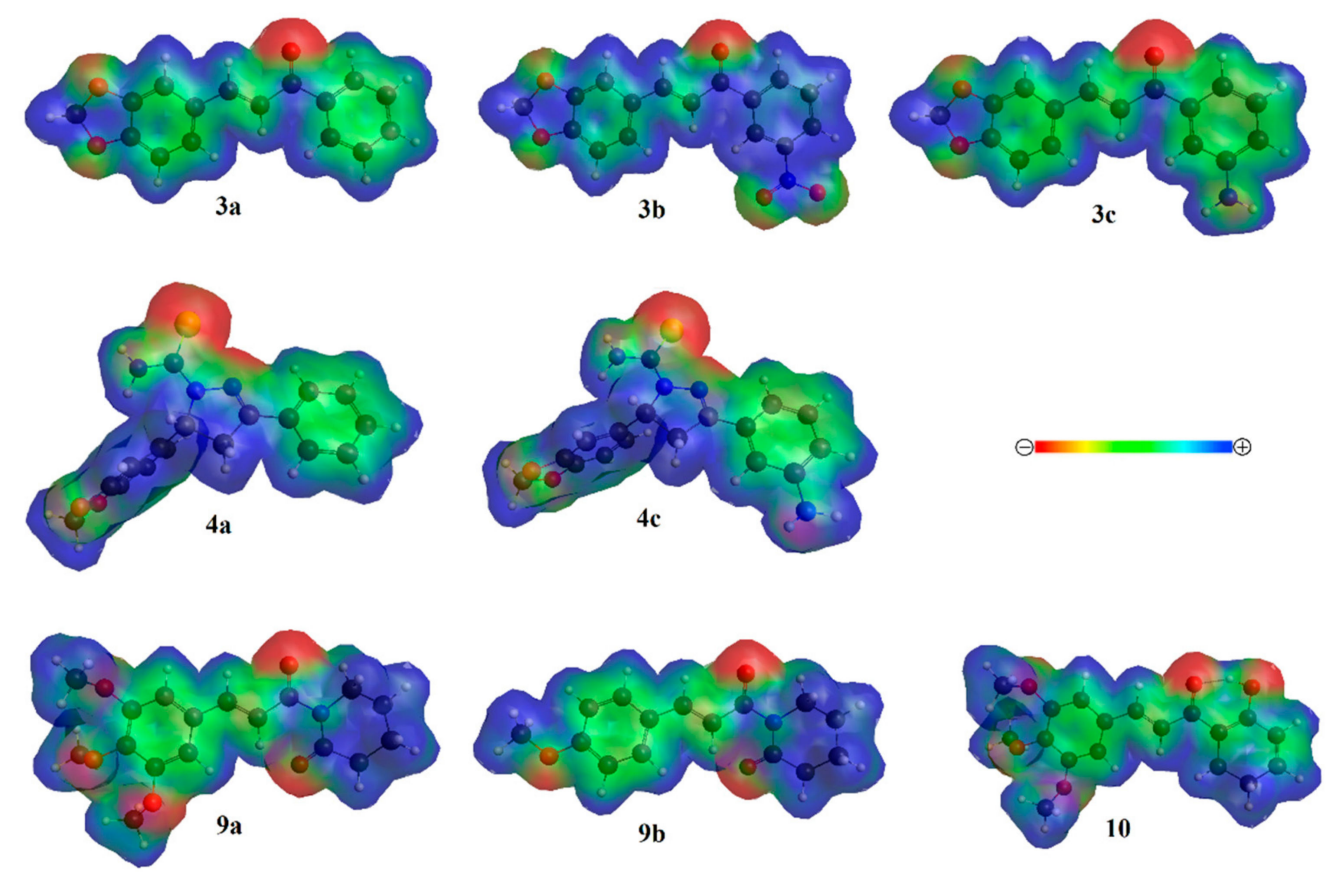
Map of electronic potential (MEP) of piperine and PPL-inspired molecules, where the scale is from negative density charge (represented by red) to positive density charge (represented by blue).

Figure 1 shows the structures resulting from *ab initio* calculations of their respective map of electrostatic potential (MEP). **3a,b,c** presented a planar structure due to the high number of conjugated double bonds. A similar result was found for piperine previously [21]. **3a**,**b,c** have the most negative charge density around oxygen atoms, while the most positive charge densities are concentrated on the extremities of the structures with the intermediate region being more neutral. As a special case, the nitro group in **3b** relocated the negative charges from the aromatic ring to the oxygen atoms, consequently the aromatic ring of **3b** became more positive when compared to **3a,c** and may explain its unreactivity to thiosemicarbazide. This relocation of negative charges caused **3b** to have high affinity for a microenvironment mainly composed by polar amino acids, which differs from **3a,c,** which preferred less polar microenvironments (see molecular docking). The MEP of **4a,c** indicated that the negative density charge was concentrated around the oxygen, nitrogen and the sulfur atoms, while the positive density charge was concentrated on the dihydropyrazole and 1,3-benzodioxole rings, with the neutral region being the aromatic ring.

A minimal puckering of the five-membered ring system of **4a** modelled in CDCl_3_ revealed a maximal torsional angle of +4.8° around C(10)-N(14)-N(13)-C(12) and a minimal torsional angle of −3.0° across the ring system. This deviation away from planarity may explain the low 15 ppm ^15^N NMR signal observed in this ring system.

**9a**, **10** and **9b** presented a planar structure, except for methoxy groups (see Figure S39, Supplementary Materials). ^1^H NMR of **10** (Figure S38, Supplementary Materials) showed a resonance peak at 15.76 ppm, associated with the hydroxyl from cyclohexanone forming an intramolecular hydrogen bond with the adjacent carbonyl oxygen; consequently, this hydrogen is not exchangeable and the signal is observed in the ^1^H NMR spectrum. According to *ab initio* calculations for **10**, the intramolecular hydrogen bond length is 1.55 Å. The planarity of the three structures are quite similar to the characteristics found in PPL [22]. According to MEP, the three structures presented a negative density charge concentrated around oxygen atoms, while the positive density distributed on the extremity of the molecule and the aromatic ring is a neutral part of the molecules. The negative density charge concentrated on the atoms identified plays a crucial role in the interaction of **10** with NF-κB, as the binding site is positively charged.

### 2.3. Molecular Docking

**Figure 2:**
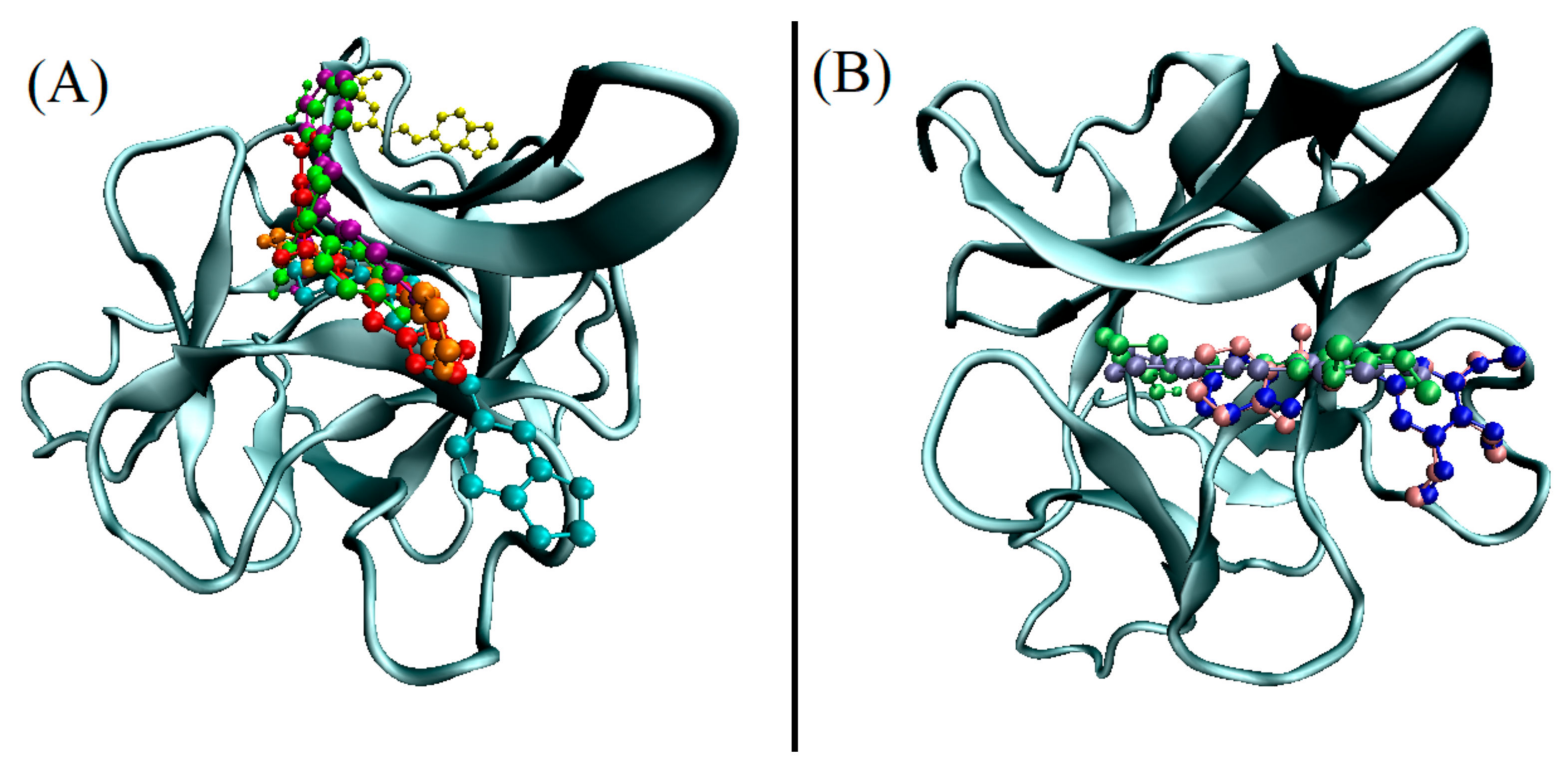
IL-1β (iceblue) binding environments to (**A**) piperine and analogues (Piperine-cyan, **3a**-orange, **4a**-purple, **3b**-yellow, **3c**-red, **4c**-green). (**B**) PPL and analogues (PPL-blue, **9a**-pink, **10**-lime, **9b**-iceblue).

According to molecular docking results with IL-1β (Figure 2), piperine and analogues **3a**, **4a**, **3c** and **4c** bound to the same binding site with the binding energy scores of −6.08, −6.04, −7.18, −6.25 and −7.22 kcal/mol, respectively.

**Figure 3:**
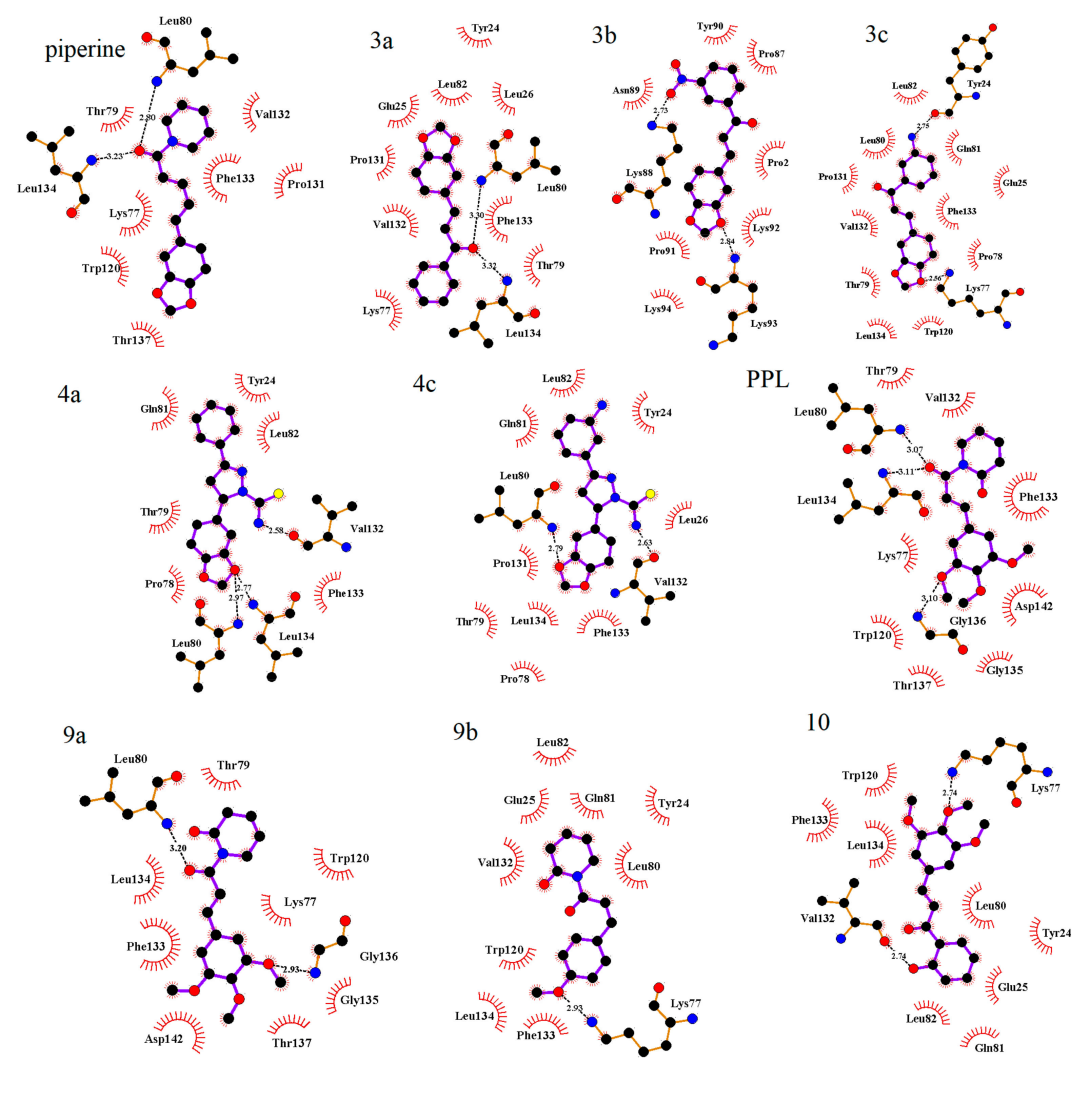
IL-1β binding microenvironments details for piperine and PPL analogues visualized with LigPlot.

Figure 3 and Table 1 show the amino acids that compose the microenvironment of each molecule with IL-1β. According to the docking results **4a**, **3c** and **4c** had a more negative binding energy than piperine; having a higher affinity for this environment when compared to piperine (Table 2). Most of the amino acids that composed the binding site are non-polar, with limited neutral and charged polar amino acids participating. The higher affinity of the analogues for the binding site revealed that the addition of an aromatic ring, and consequently the new electronic distribution, contributed to the increase in affinity. Moreover, the aromatic rings of **3a,c** exhibited cation–π interactions with the protonated amine group of Lys77 amino acid (see Supplementary Materials), which contributed to the high negative binding energy of these analogues to the protein. The cation–π interaction is caused by the molecular electron delocalization, reinforcing the importance of the increase in electron delocalization for the affinity at the IL-1β binding site.

As a consequence of the new structural properties, **4a** and **4c** presented the best affinities to this binding site. Analogue **3b** bound with a binding energy of −7.44 kcal/mol to an orthosteric binding site, where the majority of amino acids are positive and neutral polar amino acids. The differences between **3a**,**c** and **3b** are due to the aromatic ring substitution, the nitro group in **3b** drove the interaction to a less hydrophobic environment, differently from the amino group (**3c**).

The binding site found by molecular docking for PPL and analogues was the same as the piperine binding site. Table 2 shows that PPL, **9a**, **10** and **9b** had binding scores of −5.83, −5.38, −6.19 and −5.62 kcal/mol, respectively. In the case of PPL analogues, **10** presented the most negative binding energy and highest affinity for the binding site. The results showed that the removal of two methoxy groups (**9b**) limited the possibility of performing multiple hydrogen bonds. Furthermore, **9b** exhibited a cation–π interaction with the protonated amine of Lys77 (see Supplementary Materials), which contributed to the negative binding energy. Docking results also showed that the removal of the double bond in the cyclic ring (**9a**) resulted in higher binding energy (less negative) than the other molecules, which reinforced the importance of electron delocalization in the binding affinity.

**Figure 4:**
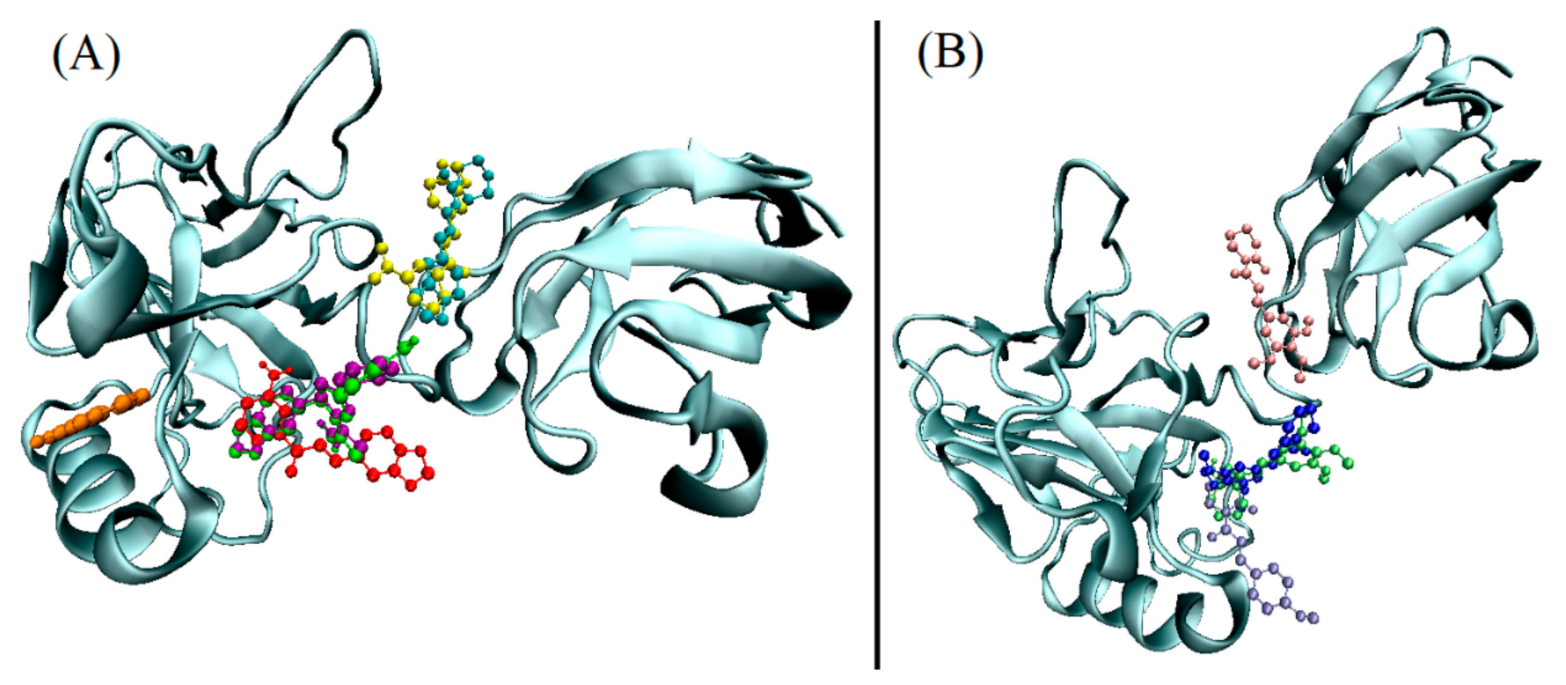
NF-κB (iceblue) binding environments to (**A**) Piperine and analogues (Piperine-cyan, **3a**-orange, **4a**-purple, **3b**-yellow, **3c**-red, **4c**-green). (**B**) PPL and analogues (PPL-blue, **9a**-pink, **10**-lime, **9b**-iceblue).

**Figure 5:**
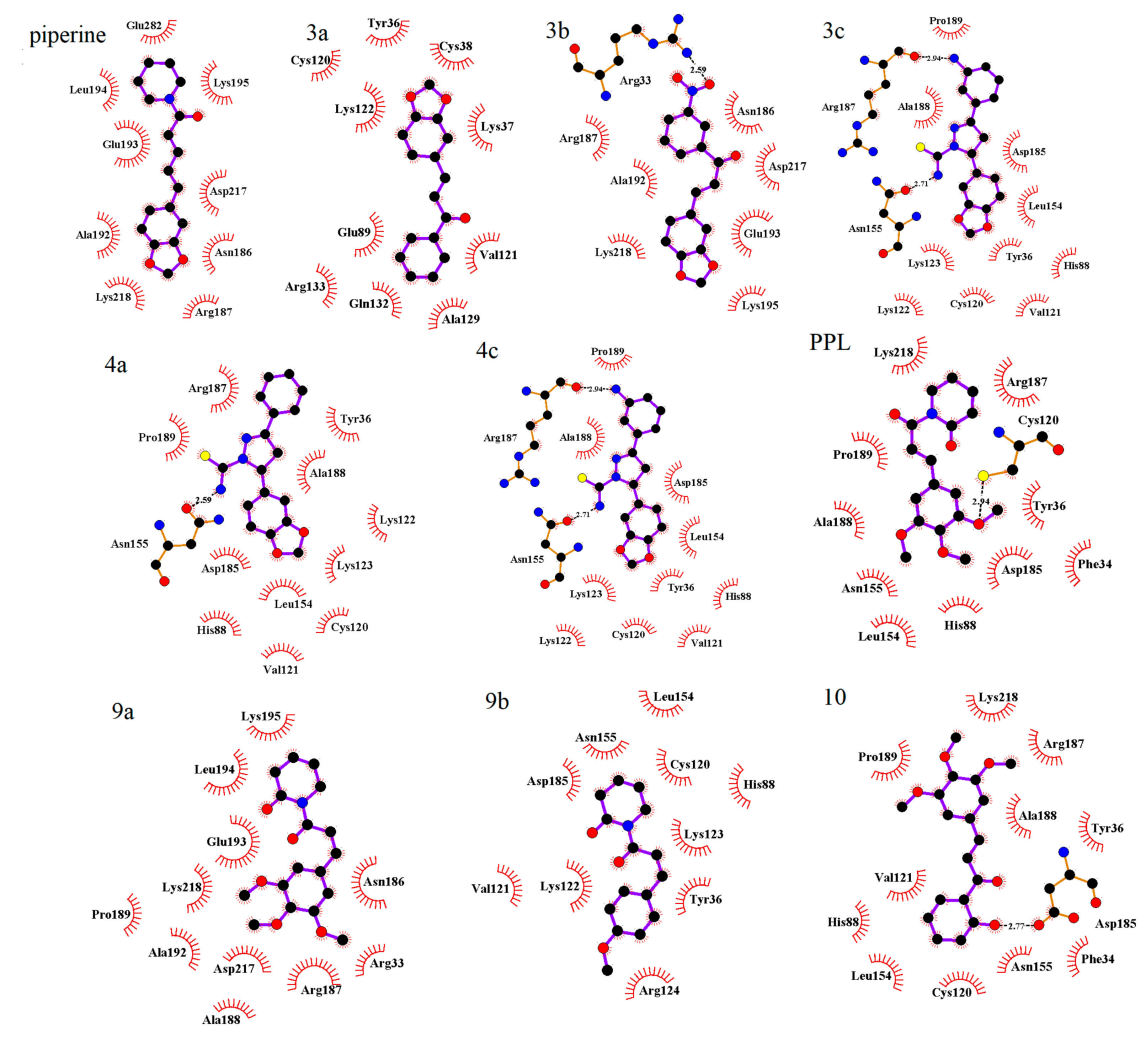
NF-κB binding microenvironments details for piperine and PPL analogues visualized with LigPlot.

In the case of the interaction with NF-κB, all the piperine and piperlongumine analogues, with the exception of **3c**, presented a more negative binding energy (Table 2) than the natural products. Piperine, **4a**, **3b**, **3c**, **4c**, PPL, **9a**, **10** and **9b** all bound to the DNA binding site of NF-κB (Figure 4) [23]. Table 3 and Figure 5 show that the microenvironments of interaction of these molecules in NF-κB present a significant number of non-polar amino acids (except for **3b**), similar to the IL-1β binding site. However, in the case of the NF-κB binding site, there is also a greater number of positively charged amino acids. The interaction of the analogues with the positively charged amino acids may play an important role in the inhibition of the interaction NF-κB(p65)-DNA, as positively charged amino acids (including Lys122 and Lys123) stabilize the NF-κB(p65)-DNA complex *via* interaction with the negatively charged phosphates groups of the DNA [24].

Chen Y.Q. et al. [24] showed that the phenol ring of Tyr36 is anchored by a hydrogen bond to the phosphate backbone of DNA and makes van der Waals contacts with the methyl group of thymine at positions 1 and 2 of DNA, which makes **3a**, **4a**, **3c**, **4c** and **9b** good candidates to inhibit this interaction. Our molecular docking results revealed that Tyr36 was part of the binding microenvironment of these analogues and performed non-specific interactions.

Moreover, the side chain of Arg187 performs a hydrogen bond with the oxygen atom of thymine, and the side chain of Arg33 performs hydrogen bonds to oxygen and nitrogen atoms of guanine [24]. In this case, our molecular docking results showed that **3b** and **4c** perform hydrogen bonds with Arg33 and Arg187, indicating promising results for these compounds as inhibitors of this interaction between the arginine and DNA bases thymine and guanine.

It has been shown that the specificity of the interaction p65-DNA is likely due to the interactions between the G-C base pair and the side chains of Arg33 and Glu39 [24]. The amino acid Arg33, highlighted as being fundamental to DNA molecular recognition, is present in the microenvironments of the analogues **3b** and **9a** and performs a hydrogen bond with **3b**, which indicates that these analogues may shield the DNA recognition.

## 3. Materials and Methods

All reagents and solvents were obtained commercially from Sigma-Aldrich or Fischer Scientific and were used as supplied, with the exception of tetrahydrofuran (THF) and dichloromethane (DCM) which were treated with 3.0 Å molecular sieves (20% *m/v*) for a minimum of 48 h prior to use [25]. The progress of the syntheses was monitored by thin-layer chromatography (TLC). General procedures for the syntheses of PPL-inspired molecules were adapted from Sun Lan-Di et al. [18] For the syntheses of piperine-inspired molecules, the procedures were adapted from Mathew, A. et al. [13].

^1^H and ^13^C NMR spectra were recorded either on a Bruker AVIII operating at 300 MHz for ^1^H and fitted with a 5mm BBFO probe or on a Bruker AVANCE NEO operating at 400 MHz for ^1^H fitted with a 5mm “smart” BBFO probe, respectively. ^1^H-^1^H COSY, ^1^H-^13^C and ^1^H-^15^N HSQC, ^1^H-^13^C and ^1^H-^15^N HMBC NMR spectra were recorded on a Bruker AVANCE NEO console operating at 500 MHz for ^1^H and fitted with a nitrogen-cooled BBFO probe. Chemical shift data for ^1^H are reported in parts per million (ppm, δ scale) downfield from tetramethylsilane (TMS:δ 0.0) and referenced internally to the residual proton in the solvent. The deuterated solvents used for NMR analysis were: chloroform (CDCl_3_: δH 7.26, δC 77.2), dimethyl sulfoxide (DMSO-*d*_6_: δH 2.50, δC 39.5), and methanol (CD_3_OD: δH 3.31, δC 49.0). Coupling constants (*J*) are given in hertz (Hz). The data are presented as follows: chemical shift, multiplicity (s = singlet, d = doublet, t = triplet, q = quartet, p = pentet, m = multiple, br = broad, app = apparent and combinations thereof), coupling constant and integration.

Mass spectra data were recorded using a Waters^®^ Xevo G2-XS TOF using electro-spray ionization in positive (ESI+) mode.

### 3.1. Synthesis of Piperine-Inspired Molecules

#### Synthesis of Piperonal (**2**)

To a stirred solution of piperonyl alcohol (**1**) (152 mg, 1.0 mmol) in DCM (100 mL), manganese dioxide (1739 mg, 20.0 mmol) was added at a rate of 1.0 mmol/h until completion. The reaction mixture was filtered through a pad of Celite^®^ and the filtrate was concentrated *in vacuo* to afford the desired compound as white crystals (148 mg, 97%). ^1^H NMR (300 MHz, CDCl_3_) δ 9.81 (s, 1H), 7.41 (dd, *J* = 7.9, 1.5 Hz, 1H), 7.33 (d, *J* = 1.5 Hz, 1H), 6.93 (d, *J* = 7.9 Hz, 1H), 6.08 (s, 2H); ^13^C NMR (101 MHz, CDCl3) δ 190.5, 153.3, 148.9, 132.1, 128.9, 108.5, 107.1, 102.3. Data were in accordance with the literature [26].

#### Synthesis of (*E*)-3-(Benzo[d][1,3]dioxol-5-yl)-1-phenylprop-2-en-1-one (**3a**)

Acetophenone (240 mg, 2.0 mmol), **2** (300 mg, 2.0 mmol), ethanol (95%, 1.0 mL) and sodium hydroxide solution (10% *w/w* in H_2_O, 1.0 mL) were added to a round-bottom flask. The reaction mixture was stirred at room temperature for 5 h. The reaction mixture was washed with ethanol (100 mL) and filtered under vacuum. The solid was dried *in vacuo* to afford the desired compound as a yellow powder (270 mg, 90%). ^1^H NMR (300 MHz, CDCl_3_) δ 8.06–7.95 (m, 2H), 7.74 (d, *J* = 15.6 Hz, 1H), 7.63–7.46 (m, 3H), 7.37 (d, *J* = 15.6 Hz, 1H), 7.19–7.08 (m, 2H), 6.85 (d, *J* = 8.0 Hz, 1H), 6.03 (s, 2H); ^13^C NMR (101 MHz, CDCl_3_) δ 190.6, 150.1, 148.6, 144.9, 138.6, 132.8, 129.5, 128.8, 128.6, 125.5, 120.3, 108.9, 106.8, 101.8; LRMS (ES^+^) *m*/*z*: 253.09 [M + H]^+^; HRMS (ES^+^) *m*/*z* calcd. C_6_H_13_O_3_ [M + H]^+^ = 253.0865; observed 253.0860 (−2.0 ppm).

#### Synthesis of (*E*)-3-(Benzo[d][1,3]dioxol-5-yl)-1-(3-nitrophenyl)prop-2-en-1-one (**3b**)

A round bottom flask was charged with 3-nitroacetophenone (330.3 mg, 2.0 mmol), **2** (300.3 mg, 2.0 mmol), ethanol (95%, 1.0 mL), and sodium hydroxide solution (10% *w/w* in H_2_O, 1.0 mL). The mixture was stirred at room temperature for 5 h. The mixture was washed with ethanol (100 mL) and filtered under vacuum. The powder was recrystallized from hot ethanol and the product was afforded as orange crystals (240 mg, 80%). ^1^H NMR (400 MHz, CDCl_3_) δ 8.82 (t, *J* = 1.9 Hz, 1H), 8.43 (ddd, *J* = 8.2, 2.3, 1.1 Hz, 1H), 8.33 (ddd, *J* = 7.8, 1.7, 1.1 Hz, 1H), 7.81 (d, *J* = 15.5 Hz, 1H), 7.71 (t, *J* = 8.0 Hz, 1H), 7.36 (d, *J* = 15.5 Hz, 1H), 7.21–7.14 (m, 2H), 6.87 (d, *J* = 8.0 Hz, 1H), 6.05 (s, 2H); ^13^C NMR (101 MHz, CDCl_3_) δ 188.0, 150.7, 148.7, 148.6, 146.8, 139.9, 134.2, 130.0, 128.9, 127.10, 126.1, 123.4, 118.7, 108.9, 106.9, 102.0; LRMS (ES^+^) *m*/*z*: 298.07 [M + H]^+^; HRMS (ES^+^) *m*/*z*: calcd. C_16_H_12_NO_5_ [M + H]^+^ = 298.0715; observed 298.0712 (1.0 ppm).

#### Synthesis of (*E*)-1-(3-Aminophenyl)-3-(benzo[d][1,3]dioxol-5-yl)prop-2-en-1-one (**3c**)

A round bottom flask was charged with 3-aminoacetophenone (270.3 mg, 2.0 mmol), **2** (300.3 mg, 2.0 mmol), ethanol (95%, 1.0 mL), and sodium hydroxide solution (10% *w/w* in H_2_O, 1.0 mL). The mixture was stirred at room temperature for 5 h. The mixture was washed with ethanol (100 mL) and filtered under vacuum. The crude product was purified by column chromatography (SiO_2_; EtOAc/hexane 1:1) to afford the product as a yellow powder (240.2 mg, 80%). ^1^H NMR (300 MHz, CDCl_3_) δ 7.73 (d, *J* = 15.6 Hz, 1H), 7.39 (ddd, *J* = 7.7, 1.7, 1.1 Hz, 1H), 7.37–7.27 (m, 3H), 7.18–7.10 (m, 2H), 6.90 (ddd, *J* = 7.9, 2.5, 1.1 Hz, 1H), 6.85 (d, *J* = 8.0 Hz, 1H), 6.04 (s, 2H), 3.85 (s, 2H); ^13^C NMR (101 MHz, CDCl_3_) δ 190.7, 150.01, 148.6, 147.0, 144.5, 139.7, 129.6, 129.6, 125.3, 120.6, 119.5, 118.9, 114.6, 108.8, 106.8, 101.8; LRMS (ES^+^) *m*/*z*: 268.10 [M + H]^+^; HRMS (ES^+^) *m*/*z*: calcd. C_16_H_14_NO_3_ [M + H]^+^ = 268.0974; observed 268.0974 (0.0 ppm).

#### Synthesis of 5-(Benzo[d][1,3]dioxol-5-yl)-3-phenyl-4,5-dihydro-1H-pyrazole-1-carbothioamide (**4a**)

Ethanol (95%, 25 mL), NaOH (1.0 g, 0.025 mol), **3a** (2.52 g, 0.01 mol) and thiosemicarbazide (911 mg, 0.01 mol) were added to a round-bottom flask. The reaction mixture was stirred for 3 h at reflux. Upon completion, the reaction mixture was added to ice. The resulting precipitate was washed with diethyl ether (100 mL) and water (100 mL) under vacuum filtration. The white powder was recrystallized from hot ethanol to afford the desired product as white crystals (1.51 g, 60%). ^1^H NMR (300 MHz, CDCl_3_) δ 7.79–7.66 (m, 2H), 7.52–7.37 (m, 3H), 7.09 (s, 1H), 6.86–6.57 (m, 3H), 6.31–5.76 (m, 4H), 3.81 (dd, *J* = 17.7, 11.4 Hz, 1H), 3.18 (dd, *J* = 17.8, 3.6 Hz, 1H); ^13^C NMR (126 MHz, CDCl_3_) δ 176.9, 156.2, 148.3, 147.2, 135.9, 131.3, 130.8, 129.1, 127.1, 119.2, 108.7, 106.1, 101.3, 63.5, 43.4; ^15^N NMR (51 MHz, CDCl_3_) δ 194.9; 101.3; 13.3; LRMS (ES^+^) *m*/*z*: 326.10 [M + H]^+^; HRMS (ES^+^) *m*/*z*: calcd. C_17_H_16_N_3_O_2_S [M + H]^+^ = 326.0963; observed 326.0968 (1.5 ppm).

#### Synthesis of 3-(3-Aminophenyl)-5-(benzo[d][1,3]dioxol-5-yl)-4,5-dihydro-1H-pyrazole-1-carbothioamide (**4c**)

A round-bottom flask was charged with ethanol (95%, 25 mL) NaOH (1000 mg, 0.025mol,), **3c** (2673 mg 0.01 mol) and thiosemicarbazide (911 mg, 0.01 mol). The reaction mixture was stirred for 3 h at reflux. Upon completion, the reaction mixture was added to ice. The resulting solid was washed with diethyl ether and water, then filtered. The crude product was purified by column chromatography (SiO_2_; EtOAc/hexane 7:3) to afford the desired compound as an off-yellow powder (1604 mg, 60%). ^1^H NMR (500 MHz, DMSO-*d*_6_) δ 7.98 (s, 1H), 7.67 (s, 1H), 7.08 (app t, *J* = 7.8 Hz, 1H), 7.03 (d app t, *J* = 7.8, 1.1 Hz, 1H), 6.99 (app t, *J* = 2 Hz,1H), 6.83 (d, *J* = 8.0 Hz, 1H), 6.67 (ddd, *J* = 7.9, 2.3, 1.1 Hz, 1H), 6.63 (d, *J* = 1.7 Hz, 1H), 6.59 (dd, *J* = 8.1, 1.8 Hz, 1H), 5.99–5.95 (m, 2H), 5.81 (dd, *J* = 11.2, 3.1 Hz, 1H), 5.18 (s, 2H), 3.81 (dd, *J* = 17.9, 11.3 Hz, 1H), 2.98 (dd, *J* = 17.8, 3.2 Hz, 1H); ^13^C NMR (126 MHz, DMSO-*d*_6_) δ 175.9, 155.8, 148.8, 147.2, 146.1, 136.9, 131.3, 129.1, 118.3, 116.2, 114.8, 112.0, 108.2, 105.8, 100.9, 62.4, 42.6; ^15^N NMR (51 MHz, CDCl_3_) δ 192.6; 108.6; 60.9; 14.6; LRMS (ES^+^) *m*/*z*: 341.11 [M + H]^+^; HRMS (ES^+^) *m*/*z*: calcd. C_17_H_17_N_4_O_2_S [M + H]^+^ = 341.1072; observed 341.1076 (1.2 ppm).

### 3.2. Synthesis of PPL-Inspired Molecules

#### Synthesis of (*E*)-3-(3,4,5-Trimethoxyphenyl)acrylic acid (**7a**)

Malonic acid (1.24 g, 12mmol) was added to a stirred solution of **6a** (1.94 g, 10 mmol), pyridine (6.0 mL) and piperidine (0.6 mL). The reaction was stirred at reflux for 4 h. The mixture was then cooled to room temperature and poured into a solution of HCl (10 M in H_2_O, 80 mL) at 0 °C. The white solid that formed was recovered by filtration and washed with water (200 mL) to afford the title compound as yellow powder (1.65g, 85%). ^1^H NMR (300 MHz, CDCl_3_) δ 7.71 (d, *J* = 15.8 Hz, 1H), 6.78 (s, 2H), 6.36 (d, *J* = 15.9 Hz, 1H), 3.90 (s, 6H), 3.89 (s, 3H). ^13^C NMR (101 MHz, CDCl_3_) δ 172.4, 153.6, 147.3, 140.7, 129.7, 116.6, 105.7, 61.2, 56.4; LRMS (ES^-^) *m*/*z*: 237.0763 [M-H]^+^. Data was in accordance with the literature [27].

#### Synthesis of (*E*)-3-(4-Methoxyphenyl)acrylic acid (**7b**)

Malonic acid (1.24 g, 12 mmol) was added to a stirred solution of **6b** (1.36 g, 10 mmol), pyridine (6 mL) and piperidine (0.6 mL). The reaction was stirred at reflux for 4 h. The mixture was then cooled to room temperature and poured into a solution of HCl (10 M in H_2_O, 80 mL) at 0 °C. The white solid that formed was recovered by filtration and washed with water (200 mL) to afford the title compound as white powder (1.33g, 98%). ^1^H NMR (300 MHz, CD_3_OD) δ 7.62 (d, *J* = 16.0 Hz, 1H), 7.58–7.47 (m, 2H), 7.01–6.88 (m, 2H), 6.33 (d, *J* = 15.9 Hz, 1H), 3.82 (s, 3H). Data were in accordance with the literature [28].

#### Synthesis of (*E*)-3-(3,4,5-Trimethoxyphenyl)acryloyl chloride (**8a**)

To a stirred solution of **7a** (714 mg, 3.0 mmol) in DCM (6.0 mL), thionyl chloride (0.65 mL, 9.0 mmol) and DMF (0.01 mL) were added. The reaction was stirred at reflux for 5 h under argon. The solvent was removed under reduced pressure and dried *in vacuo* to afford the title compound as a yellow powder (707 mg, 99%) and used immediately in the next step. ^1^H NMR (300 MHz, CDCl_3_) δ 7.75 (d, *J* = 15.4 Hz, 1H), 6.79 (s, 2H), 6.55 (d, *J* = 15.4 Hz, 1H), 3.91 (s, 3H), 3.91 (s, 6H).

#### Synthesis of (*E*)-3-(4-Methoxyphenyl)acryloyl chloride (**8b**)

To a stirred solution of **7b** (534 mg, 3.0 mmol) in DCM (6.0 mL), thionyl chloride (0.65 mL, 9.0 mmol) and DMF (0.01 mL) were added. The reaction was stirred at reflux for 5 h under argon. The solvent was removed under reduced pressure and dried *in vacuo* to afford the title compound as a yellow powder (513 mg, 96%) and used immediately in the next step. ^1^H NMR (300 MHz, CD_3_OD) δ 7.62 (d, *J* = 16.0 Hz, 1H), 7.58–7.47 (m, 2H), 7.00–6.89 (m, 2H), 6.33 (d, *J* = 15.9 Hz, 1H), 3.82 (s, 3H).

#### Synthesis of (*E*)-1-(3-(3,4,5-Trimethoxyphenyl)acryloyl)piperidin-2-one (**9a**)

To a stirred solution of δ-valerolactam (278 mg, 2.8 mmol) in THF (12 mL) at 0 °C, NaH (112 mg, 2.8 mmol, 60% *w/w* in oil) was added slowly. The reaction was warmed to room temperature and stirred for 2 h. The mixture was cooled to 0°C and **8a** (641 mg, 2.5mmol) was added. The reaction was stirred for 1 h at 0 °C and for a further 24 h at room temperature under argon. The solution was poured into ice water and stirred for 15 min. The organic phase was recovered by extraction with ethyl acetate (15 mL), washed with brine (30 mL) and NaOH (2.0 M in H_2_O, 30 mL) and dried (MgSO_4_). The organic phase was concentrated by rotary evaporation under reduced pressure. The crude product was purified by column chromatography (SiO_2_; EtOAc/petroleum ether 1:1) to afford the title compound as a yellow powder (219 mg, 34%). ^1^H NMR (300 MHz, CDCl_3_) δ 7.63 (d, *J* = 15.5 Hz, 1H), 7.35 (d, *J* = 15.6 Hz, 1H), 6.78 (s, 2H), 3.88 (s, 6H), 3.86 (s, 3H), 3.79 (td, *J* = 5.1, 4.1, 2.7 Hz, 2H), 2.68–2.53 (m, 2H), 1.95–1.80 (m, 4H). Data were in accordance with the literature [18].

#### Synthesis of (*E*)-1-(3-(4-Methoxyphenyl)acryloyl)piperidin-2-one (**9b**)

To a stirred solution of δ-valerolactam (278 mg, 2.8 mmol) in THF (12 mL) at 0 °C, NaH (112 mg, 2.8 mmol, 60% *w/w* in oil) was added slowly. The reaction warmed to room temperature and stirred for 2 h. The mixture was cooled to 0 °C and **8b** (490 mg, 2.5mmol) was added. The reaction was stirred for 1 h at 0 °C and for a further 24 h at room temperature under argon. The solution was poured into ice water and stirred for 15 min. The organic phase was recovered by extraction with ethyl acetate (15 mL), washed with brine (30 mL) and NaOH (2.0 M in H_2_O, 30 mL) and dried (MgSO_4_). The organic phase was concentrated by rotary evaporation under reduced pressure. The crude product was purified by column chromatography (SiO_2_; EtOAc/petroleum ether 1:1) to afford the title compound as a yellow powder (114 mg, 23%). ^1^H NMR (300 MHz, CDCl_3_) δ 7.69 (d, *J* = 15.6 Hz, 1H), 7.57–7.47 (m, 2H), 7.35 (d, *J* = 15.6 Hz, 1H), 6.95–6.81 (m, 2H), 3.83 (s, 3H), 3.79 (td, *J* = 5.2, 4.2, 2.3 Hz, 2H), 2.66–2.54 (m, 2H), 1.93–1.84 (m, 4H). Data were in accordance with the literature [29].

#### Synthesis (*E*)-6-(3-(3,4,5-Trimethoxyphenyl)acryloyl)cyclohex-2-en-1-one (**10**)

To a stirred solution of cyclohex-2-enone (577 mg, 6.0 mmol) in THF (15.0 mL) at −78 °C, lithium di*iso*propylamide (0.6 mL, 6.0 mmol) was added dropwise. The solution was stirred at −78 °C for 45 min under argon. A solution of **8a** (770 mg, 3.0 mmol) in THF (15 mL) was slowly added to the initial solution and stirred for a further 1 h at −78 °C, then 1 h at 0 °C. The organic phase was recovered by extraction with ethyl acetate (15 mL), washed with brine (30 mL), and dried (MgSO_4_)_._ The crude product was purified by column chromatography (SiO_2_; EtOAc/petroleum ether 1:1) to afford the title compound as a yellow powder (228 mg, 40%). ^1^H NMR (300 MHz, CDCl_3_) δ 15.76 (t, *J* = 1.1 Hz, 1H), 7.55 (d, *J* = 15.5 Hz, 1H), 6.90–6.80 (m, 2H), 6.76 (s, 2H), 6.16 (dtd, *J* = 10.0, 2.0, 0.9 Hz, 1H), 3.90 (d, *J* = 1.0 Hz, 6H), 3.88 (d, *J* = 0.9 Hz, 3H), 2.75 (t, *J* = 7.2 Hz, 2H), 2.42 (qd, *J* = 5.3, 4.7, 2.6 Hz, 2H). Data were in accordance with the literature [29] except the signal at 15.76 ppm.

### 3.3. Electronic Structure Characterization

The molecular structures of the analogues Piperine and PPL were built in Avogadro software [30] and optimized by *ab initio* calculation. The calculations were performed using the Gamess2018 quantum mechanics package [31,32] with Hartree–Fock (HF) formalism [33] and density functional theory (DFT) [34]. 6-311+G(1d,1p) was used as the set of the bases and B3LYP as the functional [35]. The optimized geometries were determined with a Polarizable Continuum Model (PCM) solvent model [36] for H_2_O and **4a** was also optimized in CHCl_3_. The optimizations were followed by harmonic frequency calculations to obtain the vibrational, the rotational, and the translational contributions to the free energy. The electrostatic potential map and the partial charges were determined using the geodesic method [37] along with the same functional base set and solvent model used for the optimization of the structure. The structure and the normal frequency modes analyses were visualized by wxMacMolPlt software [38] to assure that the result is not a structure in a transition state.

### 3.4. Molecular Docking

The optimized structures of each analogue were obtained from Section 3.3. IL-1β structure was obtained from chain A of PDB-1ITB and p65 unit of NF-κB was obtained from the chain A of PDB-2O61.

AutoDockTools [39] software of the MGL program Tools 1.5.4 was used to prepare the proteins by adding polar hydrogen atoms and Gasteiger charges. Blind docking was performed to explore the whole IL-1β protein. The maps were generated by AutoGrid 4.2 program with a spacing of 0.4583 Å, dimension of 126×126×108 points and grid center coordinates as being 41.028, −0.369 and 12.346 for x, y and z coordinates, respectively. The NF-κB maps were generated by AutoGrid 4.2 program [39] with a spacing of 0.375 Å, dimension of 90 × 82 × 92 points and grid center coordinates as being 21.573, −12.537 and −0.788 for x, y and z coordinates, respectively. AutoDock 4.2[39] was used to investigate the protein binding site using the Lamarckian Genetic Algorithm (LGA) with a population size of 150, maximum number of generations of 27000 and energy evaluations equal to 2.5 × 10^6^. The other parameters were selected as the software default. To generate different conformations, the total numbers of runs were set to 100. The final energy scores were calculated following the equation

(1) $$\Delta G=\left(V_{bound}^{L-L}-V_{umbound}^{L-L}\right)+\left(V_{bound}^{P-P}-V_{umbound}^{P-P}\right)+\left(V_{bound}^{P-L}-V_{umbound}^{P-L}\right)+\Delta S_{conformation}.$$

The potentials utilized to perform the calculation were the following:

(2) $$V\;\left(energy\;score\right)=I.\underset{i,j}{\sum }\left(\frac{A_{ij}}{r_{ij}^{12}}-\frac{B_{ij}}{r_{ij}^{6}}\right)+J.\underset{i,j}{\sum }E\left(t\right).\left(\frac{C_{ij}}{r_{ij}^{12}}-\frac{D_{ij}}{r_{ij}^{10}}\right)+K.\underset{i,j}{\sum }\frac{q_{i}.q_{j}}{\varepsilon .r_{ij}^{2}}+\Delta W$$

The weighting constants I, *J*, E(t) and K are those optimized to calibrate the empirical free energy based on a set of experimentally characterized complexes. The first term is the Lenard–Jones potential, in which parameters A and B are taken from the Amber force field. The second term refers to the hydrogen bond in which parameters C and D are obtained to ensure a minimum energy of 5.0 kcal/mol in 1.9 Å for O-H and N-H and 1.0 kcal/mol in 2.5 Å for S-H [40]. The function E(t) provides directionality based on the angle t of the geometry of an ideal hydrogen bond. The third term is a shielded Coulomb potential for electrostatic interaction. The last term is the desolvation potential based on the volume of the atoms surrounding a given atom and sheltering of the solvent [40]. The final conformations were chosen among the most negative energies that belong to the most representative cluster and visualized by visual molecular dynamics software (VMD) [41]. The binding microenvironment was generated by LigPlot [42].

## 4. Conclusions

In this study, we have confirmed the structure of some ambiguous complex natural product -likescaffolds from the literature through *de novo* synthesis, spectroscopic analysis including ^15^N NMR techniques, and *ab initio* calculations. With the definitive structures in hand, we used molecular docking to investigate the rationale for the promising biological results these molecules exhibit in the inhibition of the inflammatory response (*via* downregulation of IL-1β and NF-κB pathway). This work revealed **3a**,**c**, **4a**,**c** and **10** as the most promising molecules to be evaluated as IL-1β and NF-κB inhibitors in future studies.

## Figures and Tables

**Table 1:** Amino acids that compose the microenvironment of the complex IL-1β/piperine and PPL analogues, obtained by molecular docking.

Molecule	Amino Acids			
	Non-Polar	Positively Charged	Negatively Charged	Polar
Piperine	Leu80, Leu134, Val32, Phe133, Pro131, Trp120	Lys77	-	Thr137, Thr79
**3a**	Leu82, Leu26, Leu80, Leu134, Phe133, Val132 and Pro131	Lys77	Glu25	Thr79
**4a**	Leu80,Leu82, Leu134,Val132, Phe133, Pro78	-	-	Thr79, Gln81, Tyr24
**3b**	Pro2, Pro87, Pro91	Lys88, Lys92, Lys93, Lys94	-	Asn89, Tyr90,
**3c**	Leu80, Leu82, Leu134, Phe133, Pro78, Trp120, Val132, Pro131	Lys77	Glu25	Tyr24, Gln81
**4c**	Leu26, Val132, Phe133, Pro78, Leu134, Leu82 Pro131, Leu80,	-	-	Gln81, Tyr24, Thr79
PPL	Val132, Phe133, Gly135, Gly136, Trp120, Leu134, Leu80	Lys77	Asp142	Thr79, Thr137
**9a**	Leu80, Trp120, Gly136, Gly135, Phe133, Leu134	Lys77	Asp142	Thr79, Thr137
**10**	Trp120, Leu80, Leu82, Val32, Leu134, Phe133	Lys77	Glu25	Tyr24, Gln81
**9b**	Leu82, Leu80, Phe133, Leu134, Trp120, Val132	Lys77	Glu25,	Gln81, Tyr24

**Table 2:** Binding energy scores obtained for the proteins IL-1β and NF-κB with Piperine, PPL and analogues.

Molecules	Energy Score kcal/mol (IL-1B)	Energy Score kcal/mol (NF-KB)
Piperine	−6.08	−6.08
**3a**	−6.04	−6.53
**3b**	−7.44	−6.24
**3c**	−6.25	−6.07
**4a**	−7.18	−6.64
**4c**	−7.22	−6.4
PPL	−5.83	−5.36
**9a**	−5.38	−5.85
**10**	−6.19	−5.72
**9b**	−5.62	−5.87

**Table 3:** Amino acids that compose the microenvironment of the complex NF-κB/piperine and PPL analogues, obtained by molecular docking.

Molecules	Amino Acids			
	Non-Polar	Positively Charged	Negatively Charged	Polar
Piperine	Ala192, Leu194	Lys195, Arg187, Lys218	Glu193, Glu282, Asp217	Asn186,
**3a**	Val121, Ala129, Cys38, Cys120	Lys37, Lys122, Arg133	Glu89	Tyr36, Gln132
**4a**	Ala188, Cys120, Leu154, Val121, Pro189	Arg187, Lys122, Lys123, His88	Asp185	Tyr36, His88, Asn155
**3b**	Ala192	Arg33, Arg187, Lys195, Lys218	Asp217, Glu193,	Asn186,
**3c**	Cys120, Leu154, Val121, Pro189, Ala188, Phe34	His88, Lys123	-	His88, Tyr36, Asn155, Gln220, Asn190, Asp185
**4c**	Pro189, Leu154, His88, Val121, Cys120, Ala188	Lys123, Lys122, Arg187	-	Asp185, Tyr36, His88, Asn155,
PPL	Cys120, Phe34, Leu154, Ala188, Pro189	Lys218, Arg187, His88	Asp185,	Tyr36, Asn155
**9a**	Ala188, Ala192, Pro189	Lys218, Lys195, Arg33, Arg187, Lys194	Asp217, Glu193	Asn186,
**10**	Ala188, Phe34, Cys120, Leu154, Val121, Pro189	Lys218, Arg187, His88	Asp185	Asn155, His88
**9b**	Leu154, Cys120, Val121,	His88, Lys123, Lys122, Arg124	Asp185	Tyr36, Asn155

## Supplementary Materials

The following are available online, copies of ^1^H, ^13^C and 2D NMR spectra, and *ab initio* calculations of selected dihedral angles.
