# Supplementary material for: Synthesis and Spectroscopic Analysis of Piperine- and Piperlongumine-Inspired Natural Product Scaffolds and Their Molecular Docking with IL-1β and NF-κB Proteins
Source: Molecules. 2020 Jun 19;25(12):2841. doi: 10.3390/molecules25122841 (PMC7356504; doi:10.3390/molecules25122841)
Supplement: Supplementary file 1 [file molecules-25-02841-s001.pdf]

ELECTRONIC SUPPORTING INFORMATION

Synthesis and Spectroscopic Analysis of *Piperine* and *Piperlongumine*-Inspired Natural Product Scaffolds and their Molecular Docking with IL-1β and NF-κB proteins

Gabriel Zazeri,[1,2] Ana Paula R. Povinelli,[1,2] Cécile S. Le Duff,[3] Bridget Tang,[3] Marinonio L. Cornelio,[1]* and Alan M. Jones[2]*

[1] Departamento de Física – IBILCE, Rua Cristovão Colombo, 2265 CEP 15054-000 São José do Rio Preto - São Paulo, Brazil

[2] School of Pharmacy, University of Birmingham, Edgbaston, B15 2TT, United Kingdom

[3] School of Chemistry, University of Birmingham, Edgbaston, B15 2TT, United Kingdom

*corresponding authors: MLC (m.cornelio@unesp.br) and AMJ (a.m.jones.2@bham.ac.uk)

CONTENTS

^{1}H NMR spectrum of **2** (300 MHz, $CDCl_3$) page S3
^{13}C NMR spectrum of **2** (101 MHz, $CDCl_3$) page S4
^{1}H NMR spectrum of **3a** (300 MHz, $CDCl_3$) page S5
^{13}C NMR spectrum of **3a** (101 MHz, $CDCl_3$) page S6
Mass Spectrometry of **3a** page S7
^{1}H NMR spectrum of **3b** (400 MHz, $CDCl_3$) page S8
^{13}C NMR spectrum of **3b** (101 MHz, $CDCl_3$) page S9
Mass Spectrometry of **3b** page S10
^{1}H NMR spectrum of **3c** (300 MHz, $CDCl_3$) page S11
^{13}C NMR spectrum of **3c** (101 MHz, $CDCl_3$) page S12
Mass Spectrometry of **3c** page S13
^{1}H NMR spectrum of **4a** (300 MHz, $CDCl_3$) page S14
^{13}C NMR spectrum of **4a** (126 MHz, $CDCl_3$) page S15
^{1}H-^{1}H COSY NMR spectrum of **4a** page S16
^{1}H-^{13}C HSQC NMR spectrum of **4a** page S17
^{1}H-^{13}C HMBC NMR spectrum of **4a** page S18
^{1}H-^{15}N HSQC NMR spectrum of **4a** page S19
^{1}H-^{15}N HMBC NMR spectrum of **4a** page S20
Mass Spectrometry of **4a** page S21
^{1}H NMR spectrum of **4c** (500 MHz, DMSO-d_6) page S22
^{13}C NMR spectrum of **4c** (126 MHz, DMSO-d_6) page S23
^{1}H-^{1}H COSY NMR spectrum of **4c** page S24
^{1}H-^{13}C HSQC NMR spectrum of **4c** page S25
^{1}H-^{13}C HMBC NMR spectrum of **4c** page S26
^{1}H-^{15}N HSQC NMR spectrum of **4c** page S27
^{1}H-^{15}N HMBC NMR spectrum of **4c** page S28
Mass Spectrometry of **4c** page S29
^{1}H NMR spectrum of **7a** (300 MHz, $CDCl_3$) page S30
^{13}C NMR spectrum of **7a** (101 MHz, $CDCl_3$) page S31
Mass Spectrometry of **7a** page S32
^{1}H NMR spectrum of **7b** (300 MHz, CD_3OD) page S33
^{1}H NMR spectrum of **8a** (300 MHz, $CDCl_3$) page S34
^{1}H NMR spectrum of **8b** (300 MHz, CD_3OD) page S35
^{1}H NMR spectrum of **9a** (300 MHz, $CDCl_3$) page S36
^{1}H NMR spectrum of **9b** (300 MHz, $CDCl_3$) page S37
^{1}H NMR spectrum of **10** (300 MHz, $CDCl_3$) page S38
Computational models page S39
Ab initio Calculation of selected dihedral torsional angles page S40
References page S48

^{1}H NMR spectrum of **2** (300 MHz, $CDCl_3$)

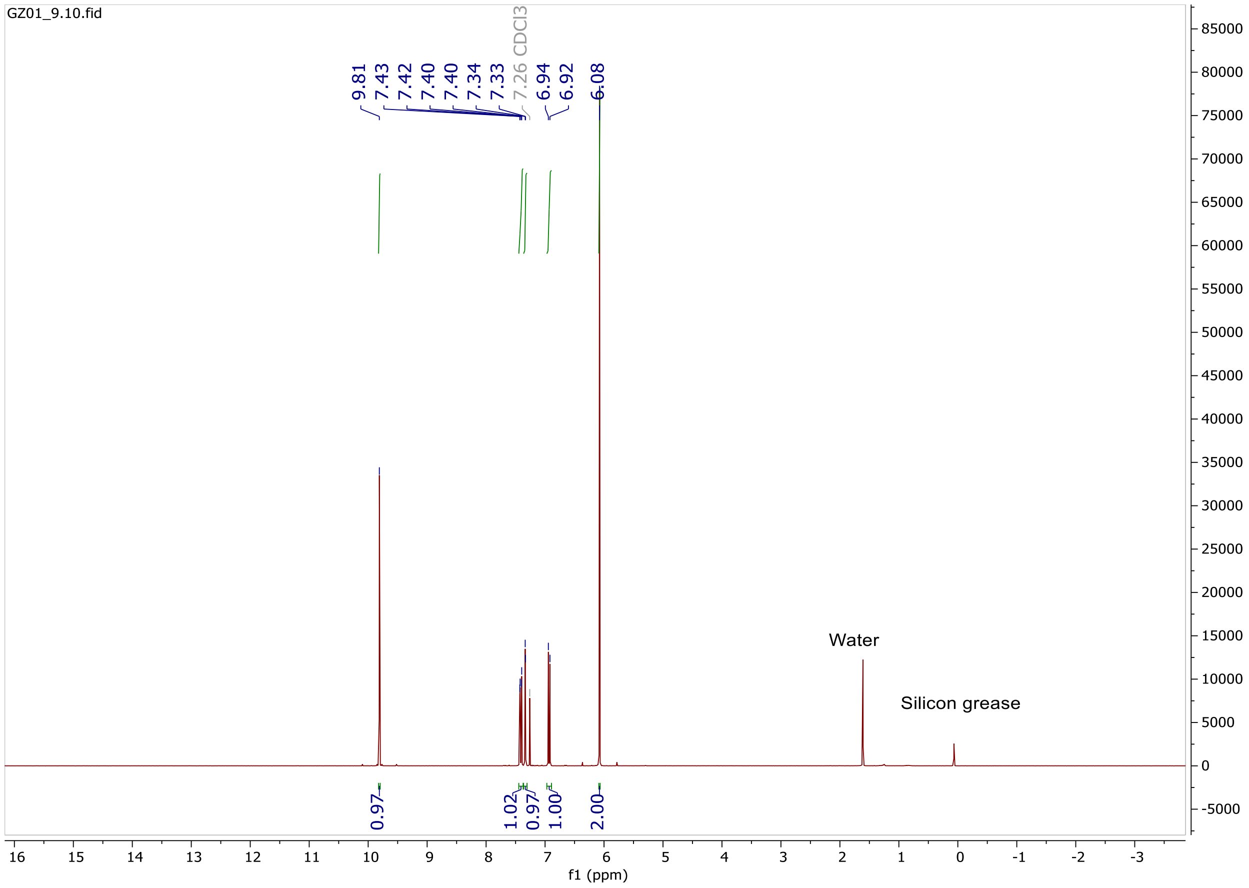

^{13}C NMR spectrum of **2** (101 MHz, $CDCl_3$)

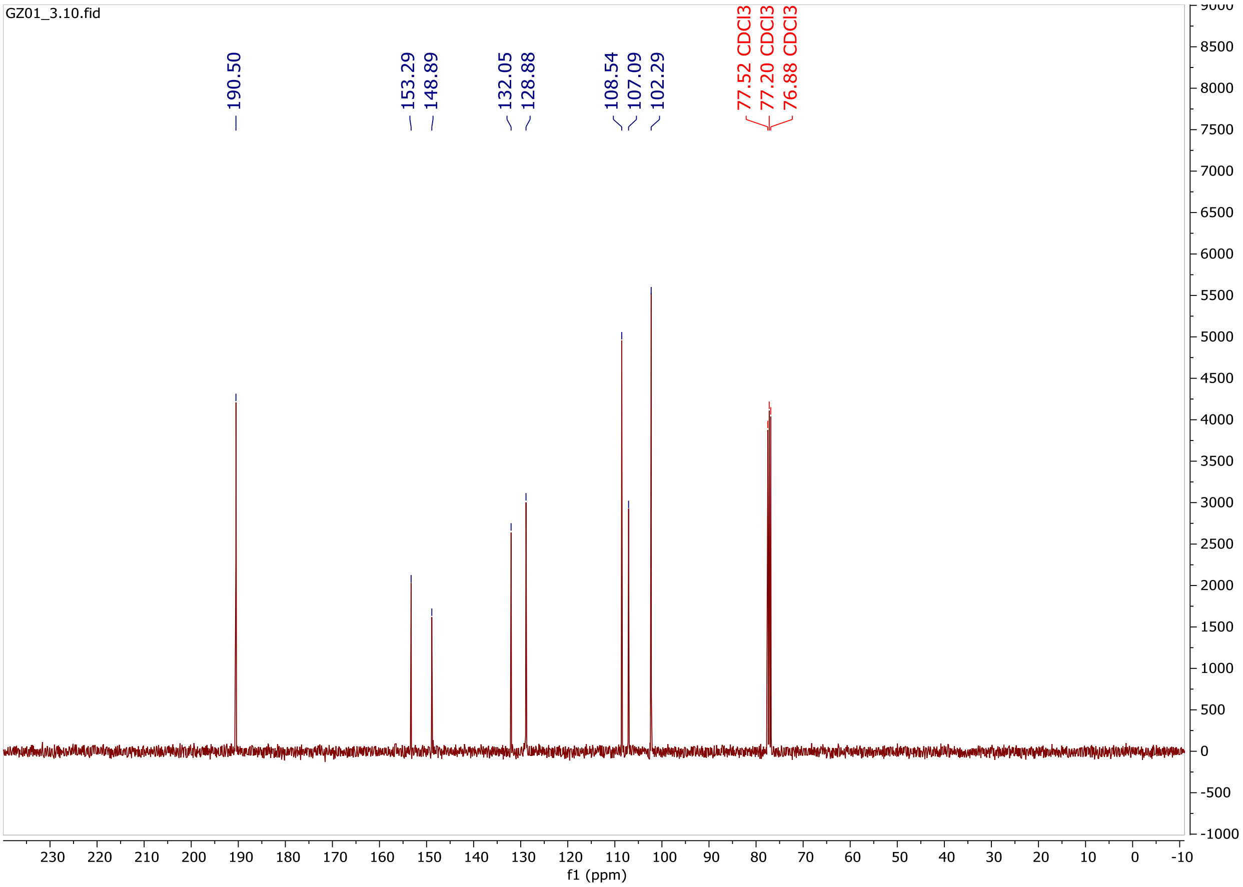

^{1}H NMR spectrum of **3a** (300 MHz, $CDCl_3$)

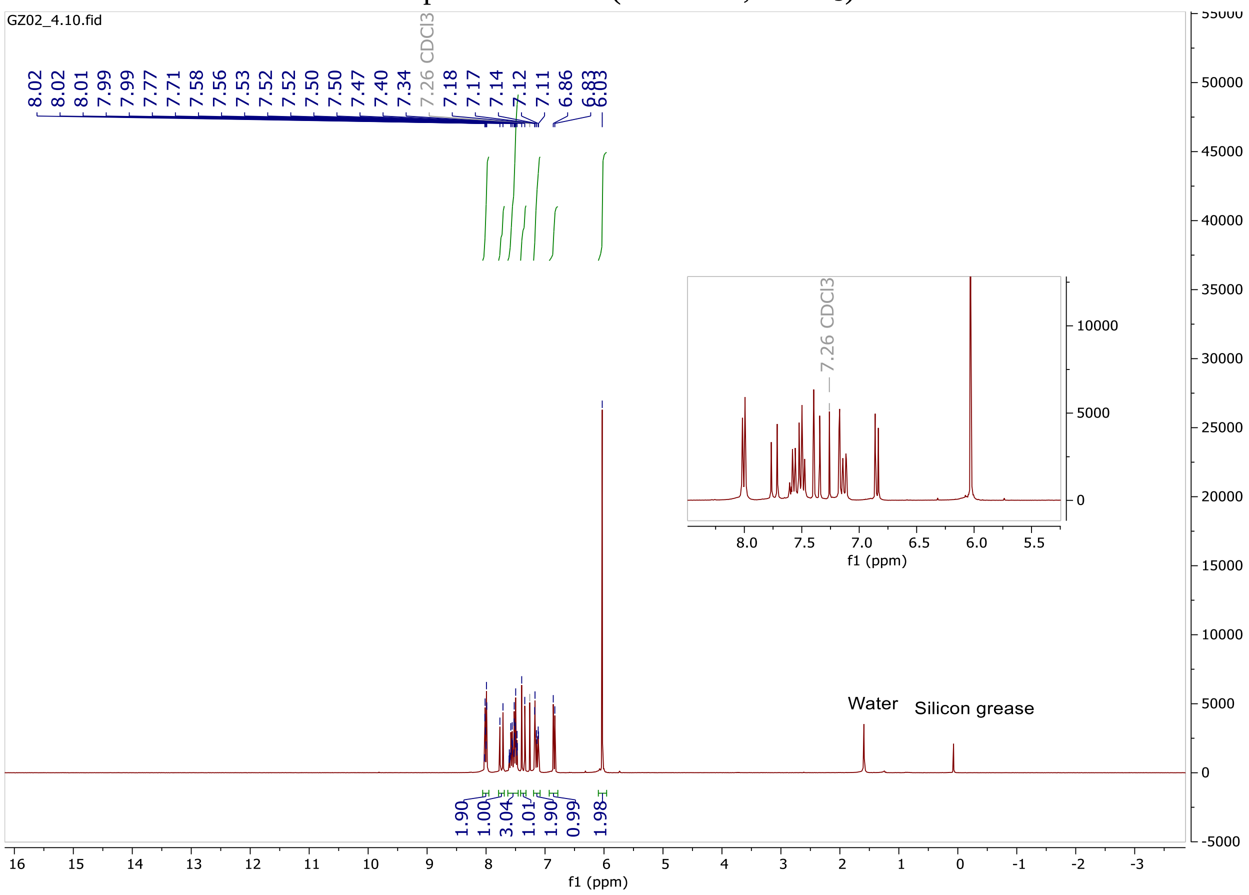

^{13}C NMR spectrum of **3a** (101 MHz, $CDCl_3$)

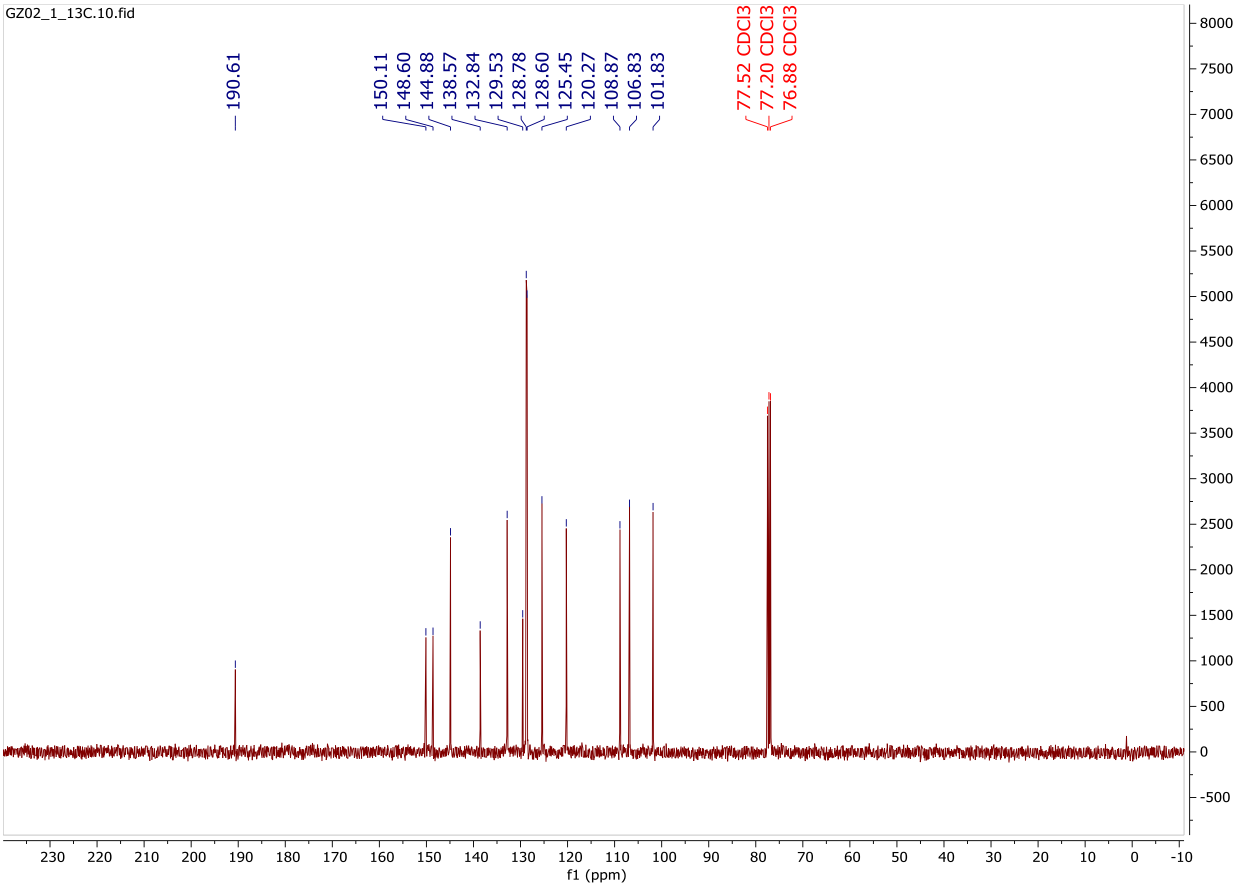

Mass Spectrometry of **3a**

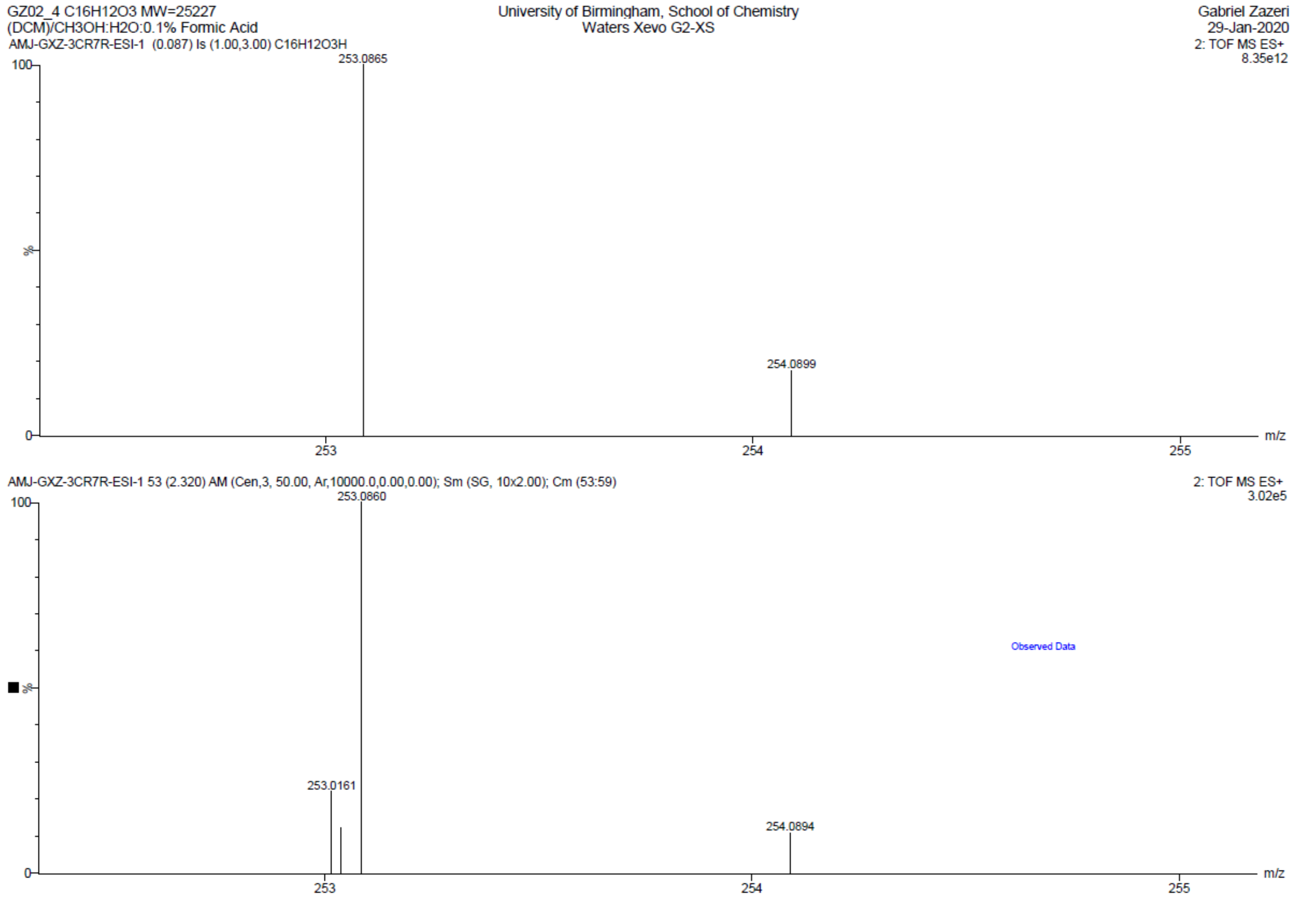

^{1}H NMR spectrum of **3b** (400 MHz, $CDCl_3$)

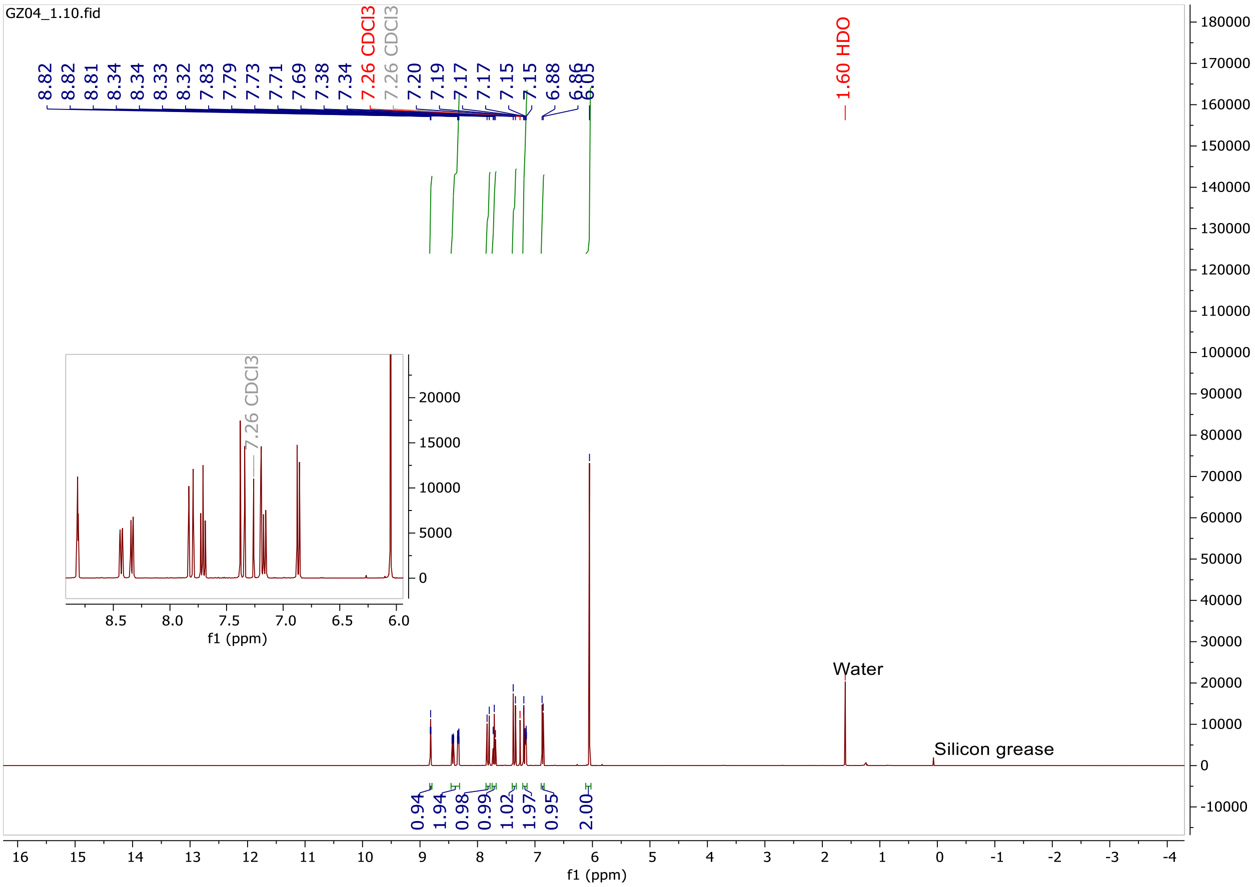

^{13}C NMR spectrum of **3b** (101 MHz, $CDCl_3$)

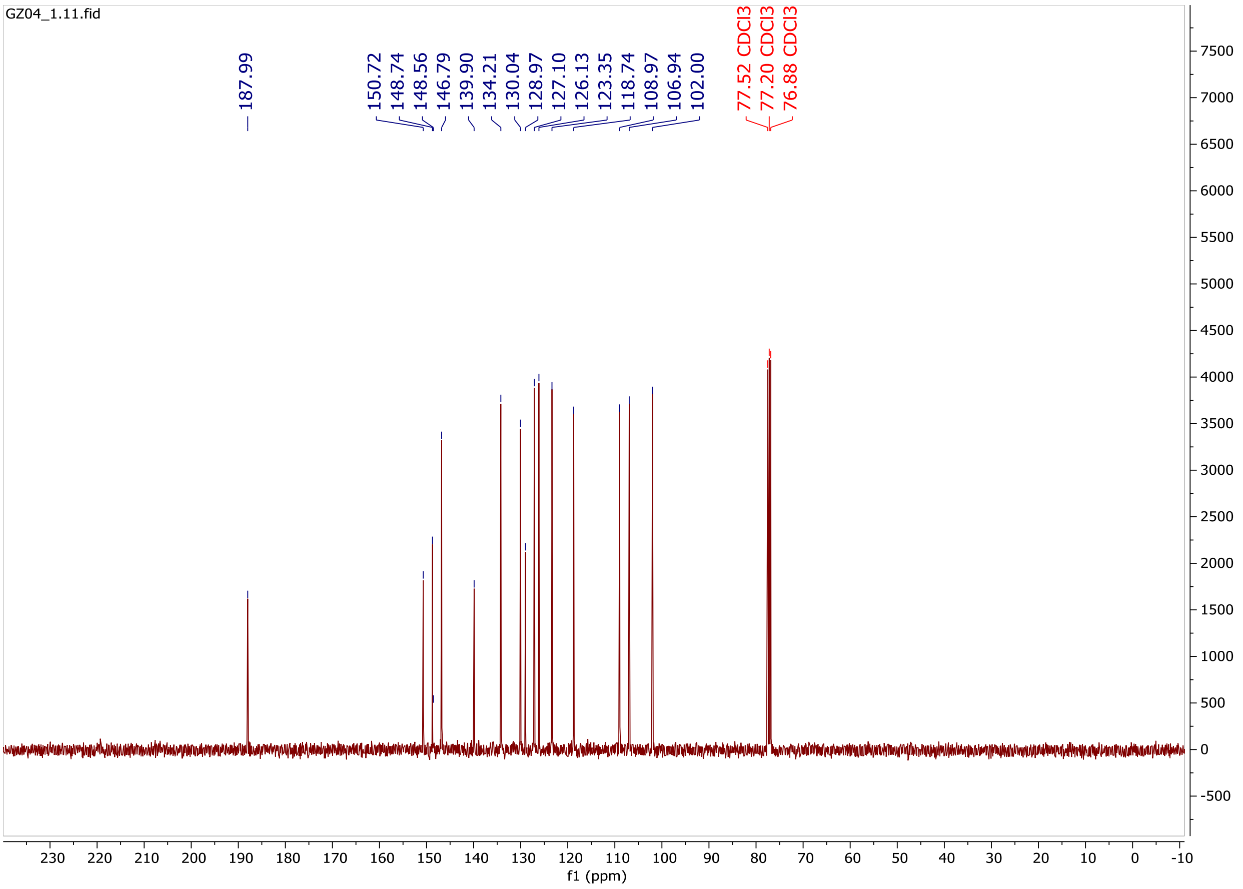

Mass Spectrometry of **3b**

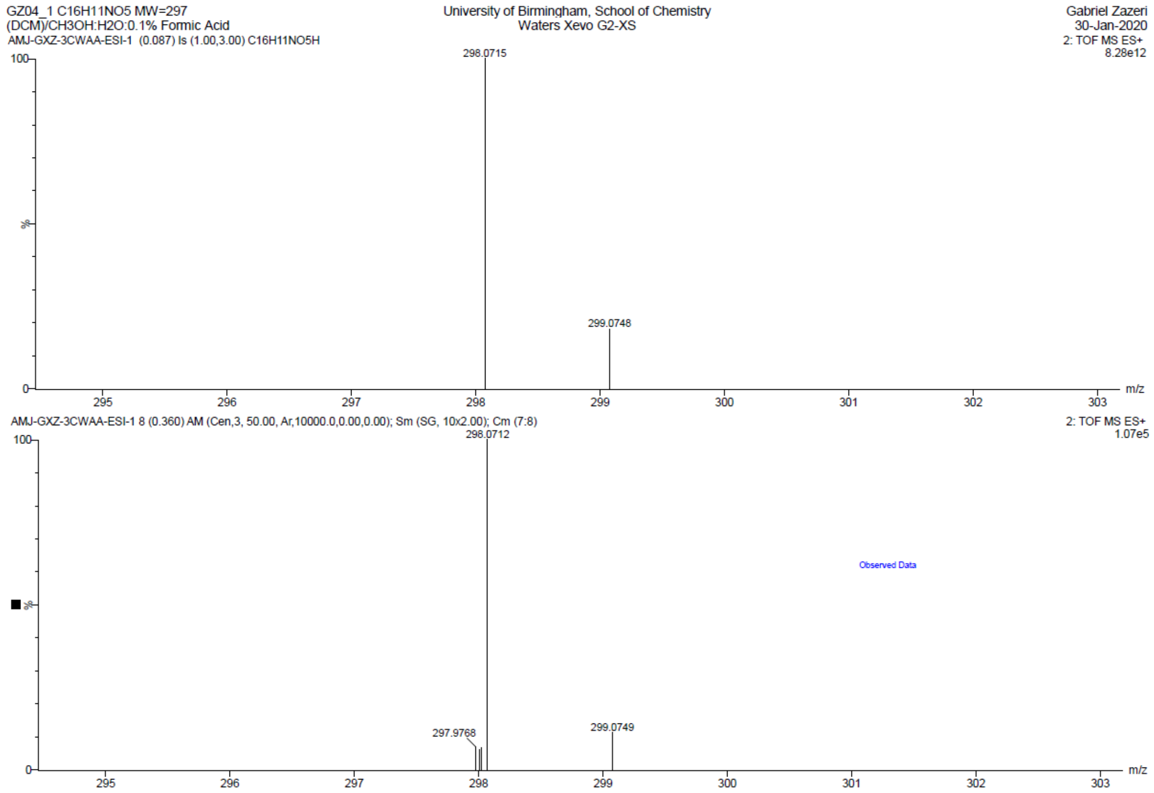

^{1}H NMR spectrum of **3c** (300 MHz, $CDCl_3$)

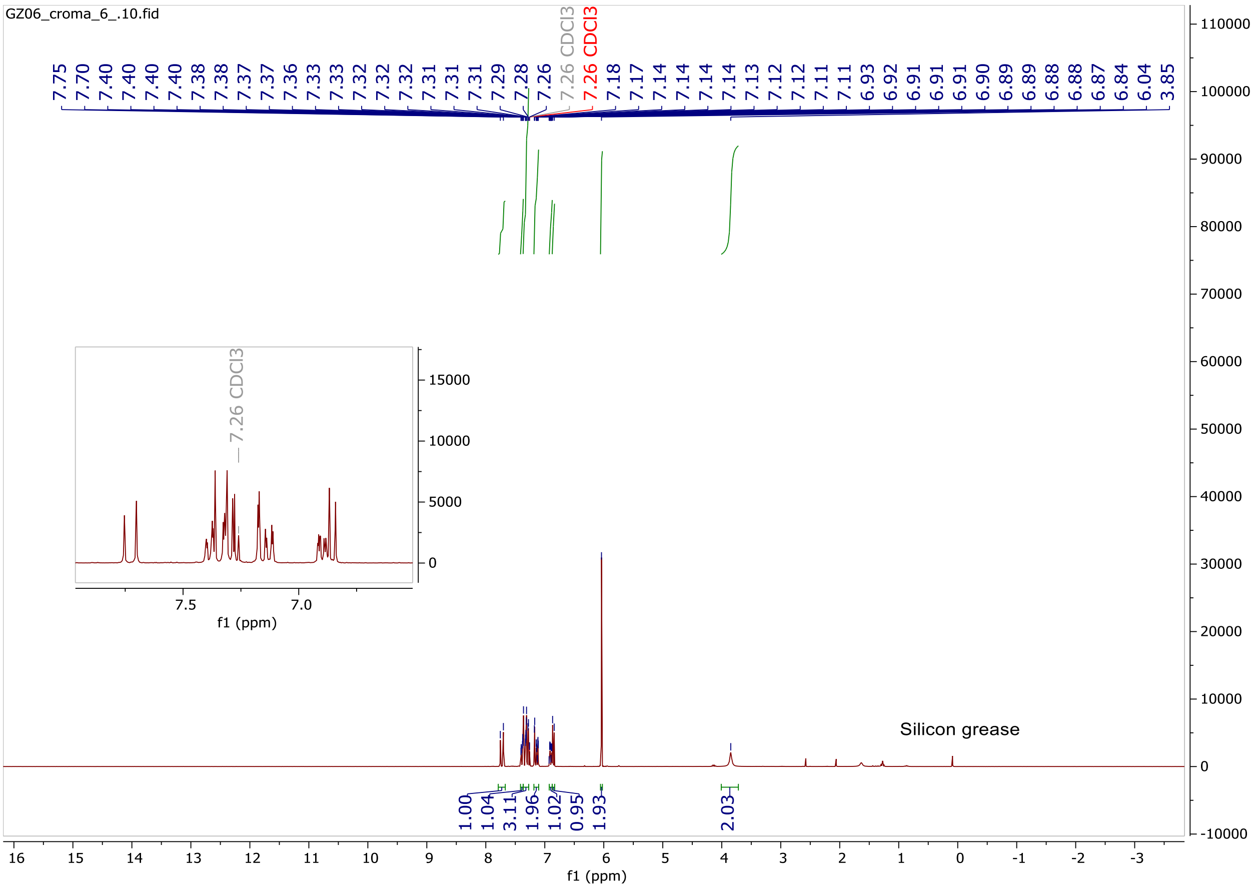

^{13}C NMR spectrum of **3c** (101 MHz, $CDCl_3$)

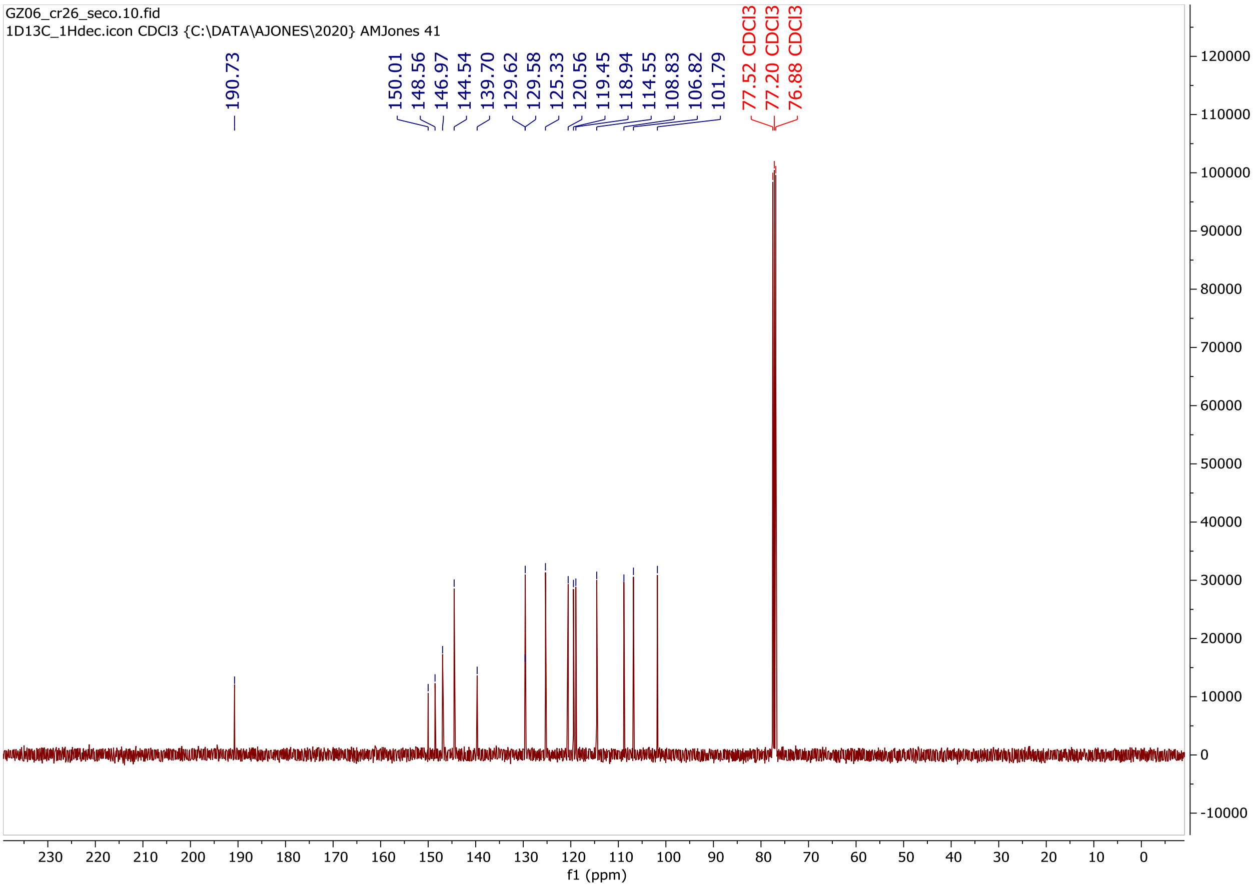

Mass Spectrometry of **3c**

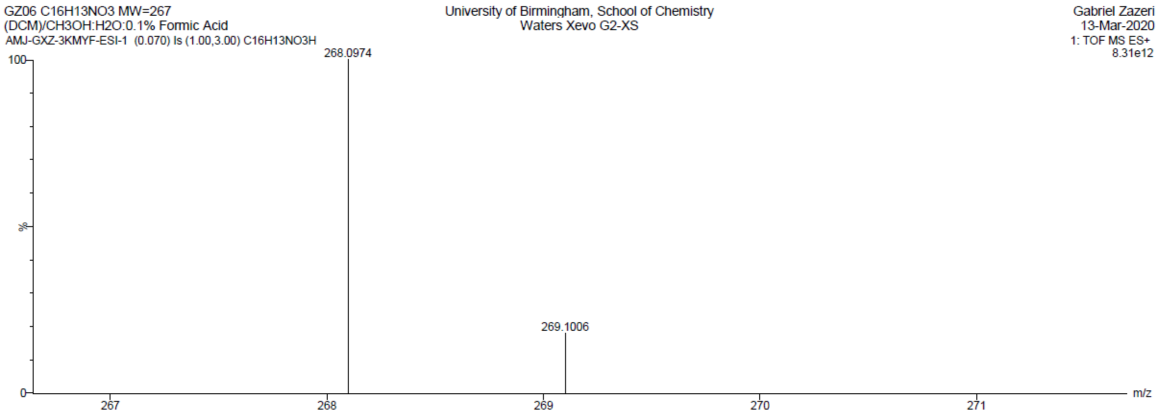

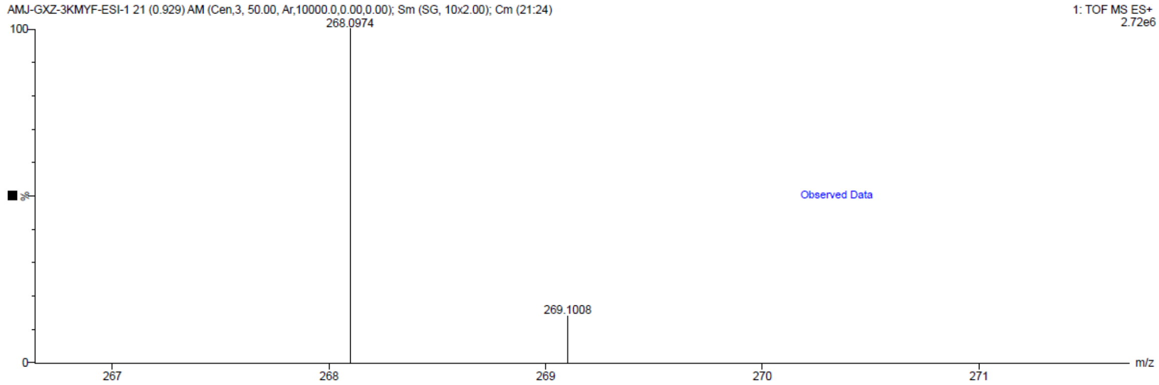

^{1}H NMR spectrum of **4a** (300 MHz, $CDCl_3$)

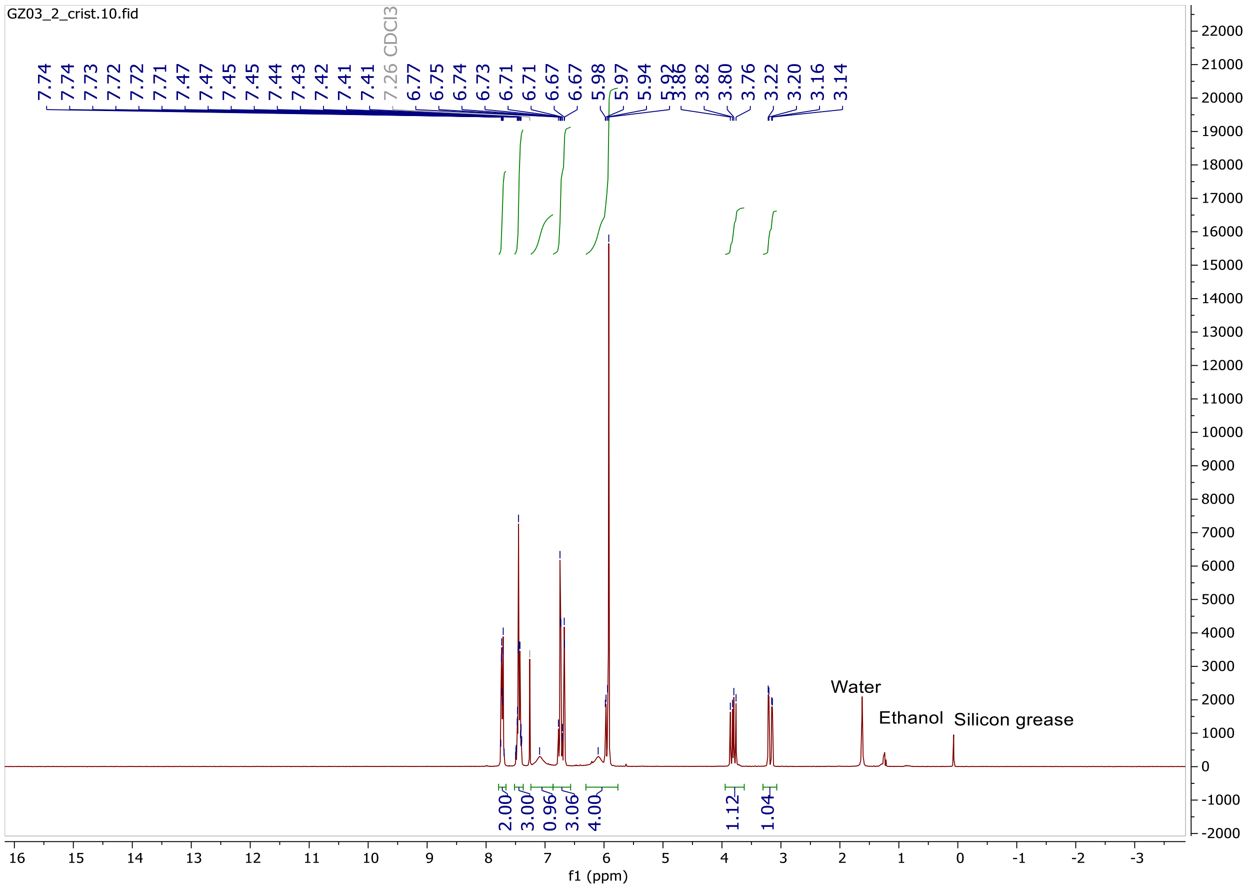

^{13}C NMR spectrum of **4a** (126 MHz, $CDCl_3$)

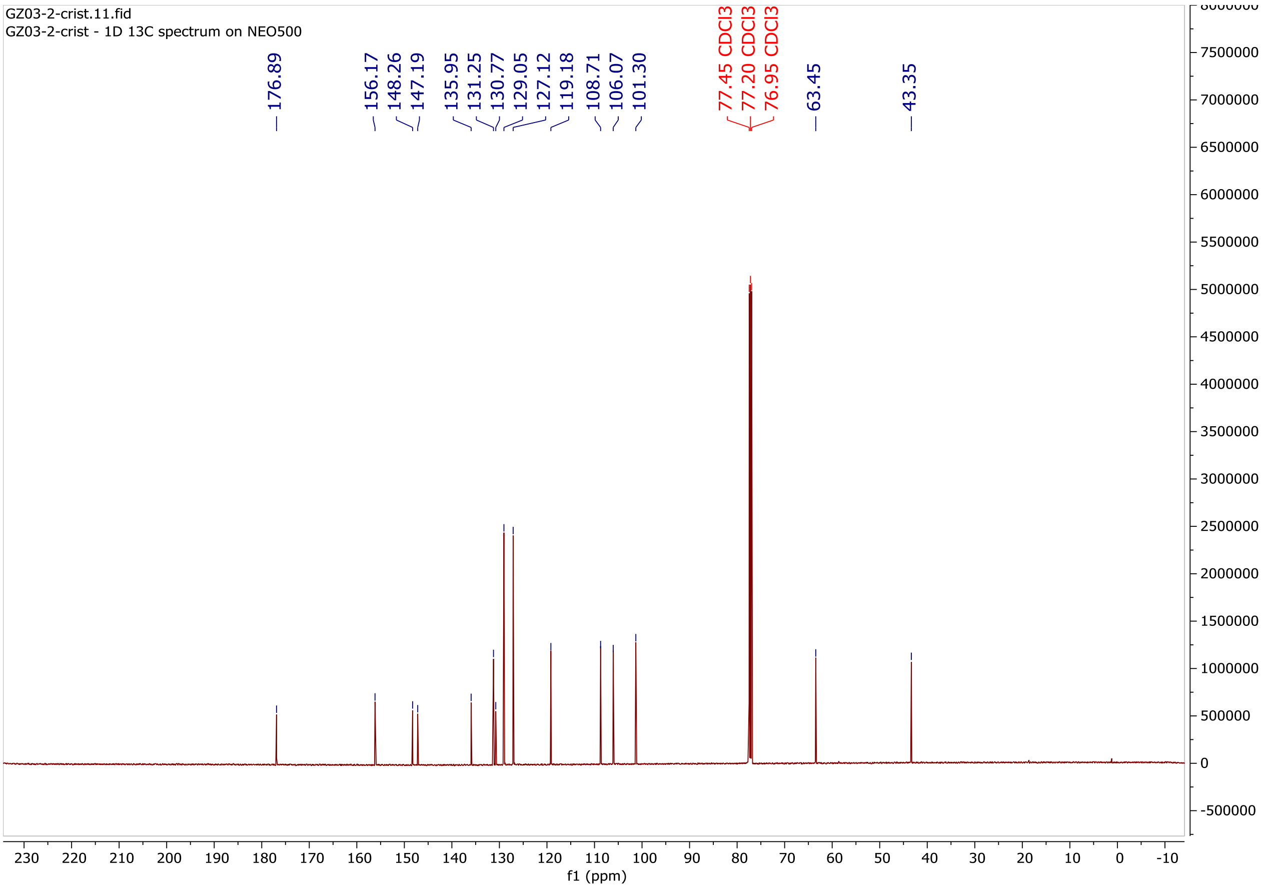

^{1}H-^{1}H COSY NMR spectrum of **4a**

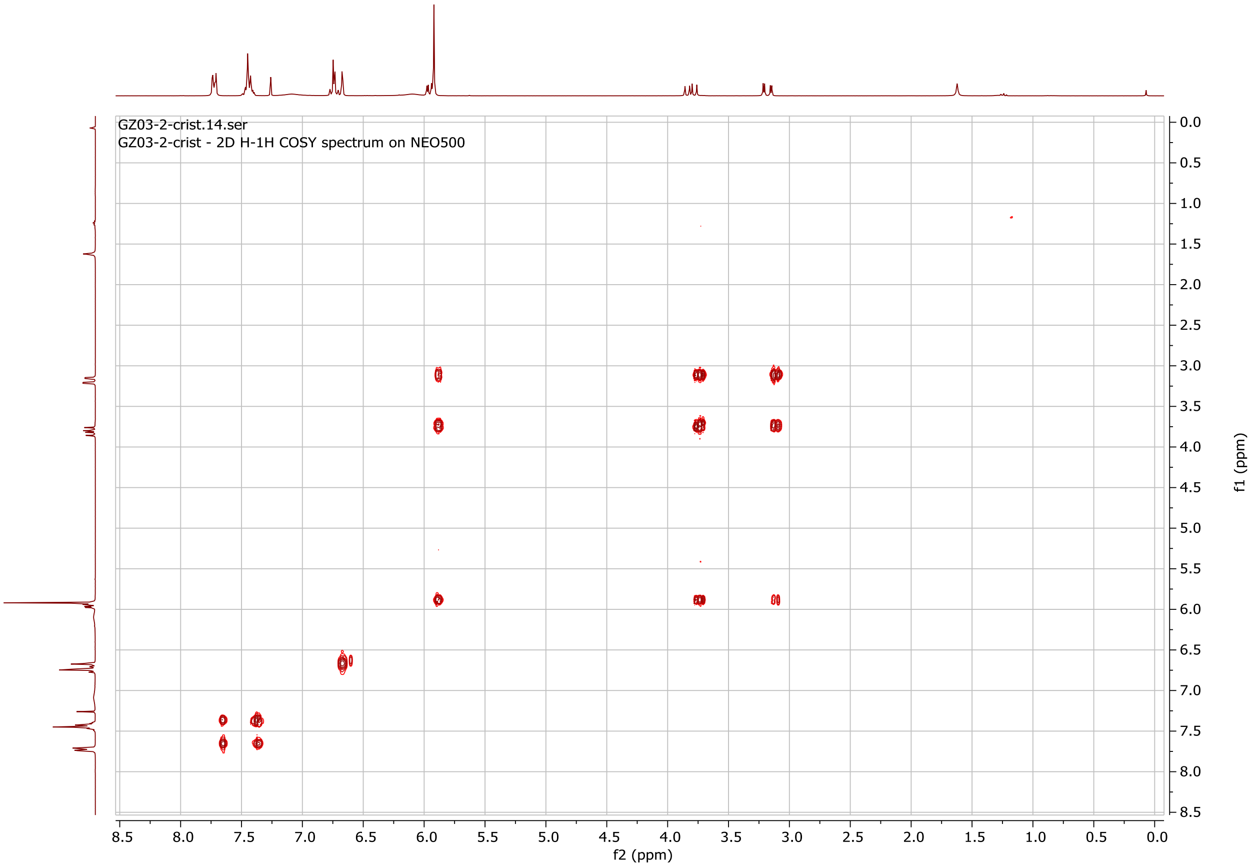

^{1}H-^{13}C HSQC NMR spectrum of **4a**

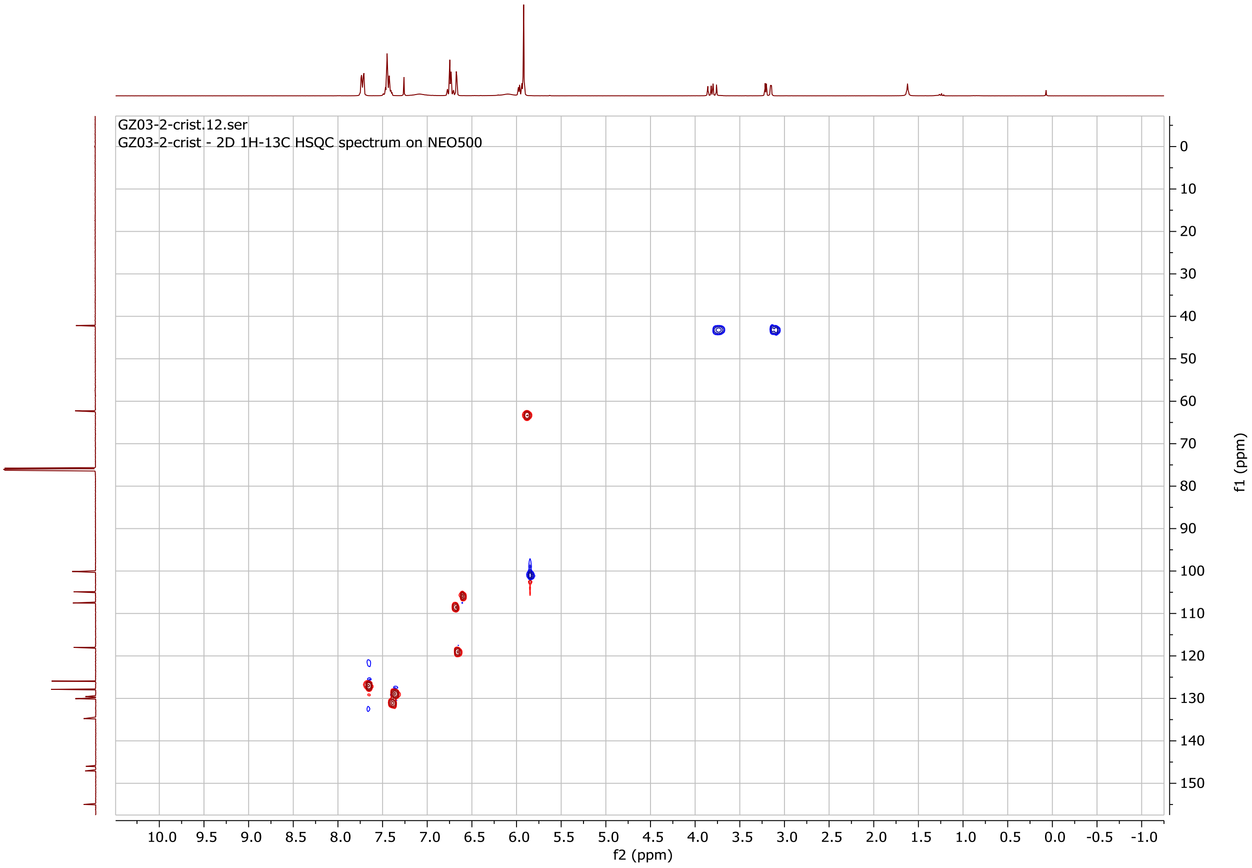

^{1}H-^{13}C HMBC NMR spectrum of **4a**

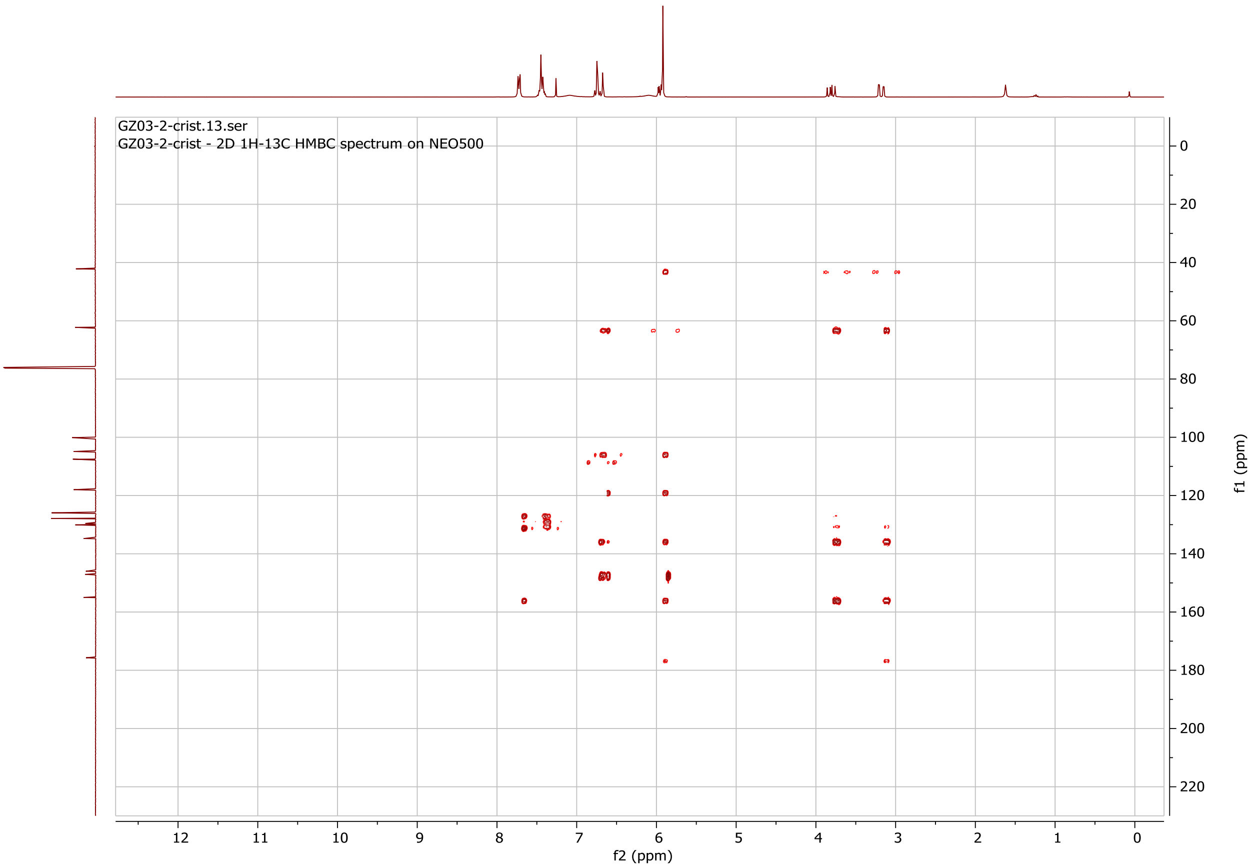

^{1}H-^{15}N HSQC NMR spectrum of **4a**

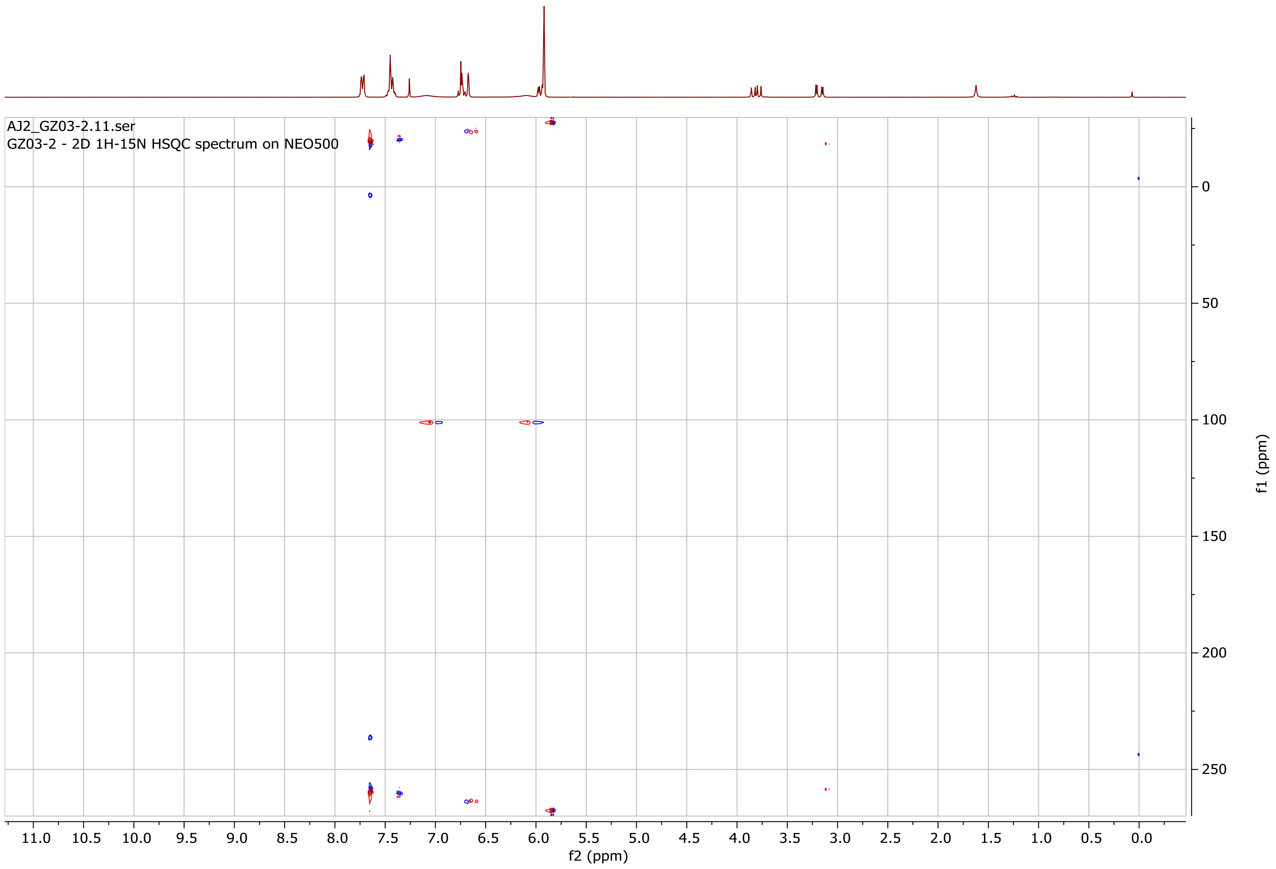

^{1}H-^{15}N HMBC NMR spectrum of **4a**

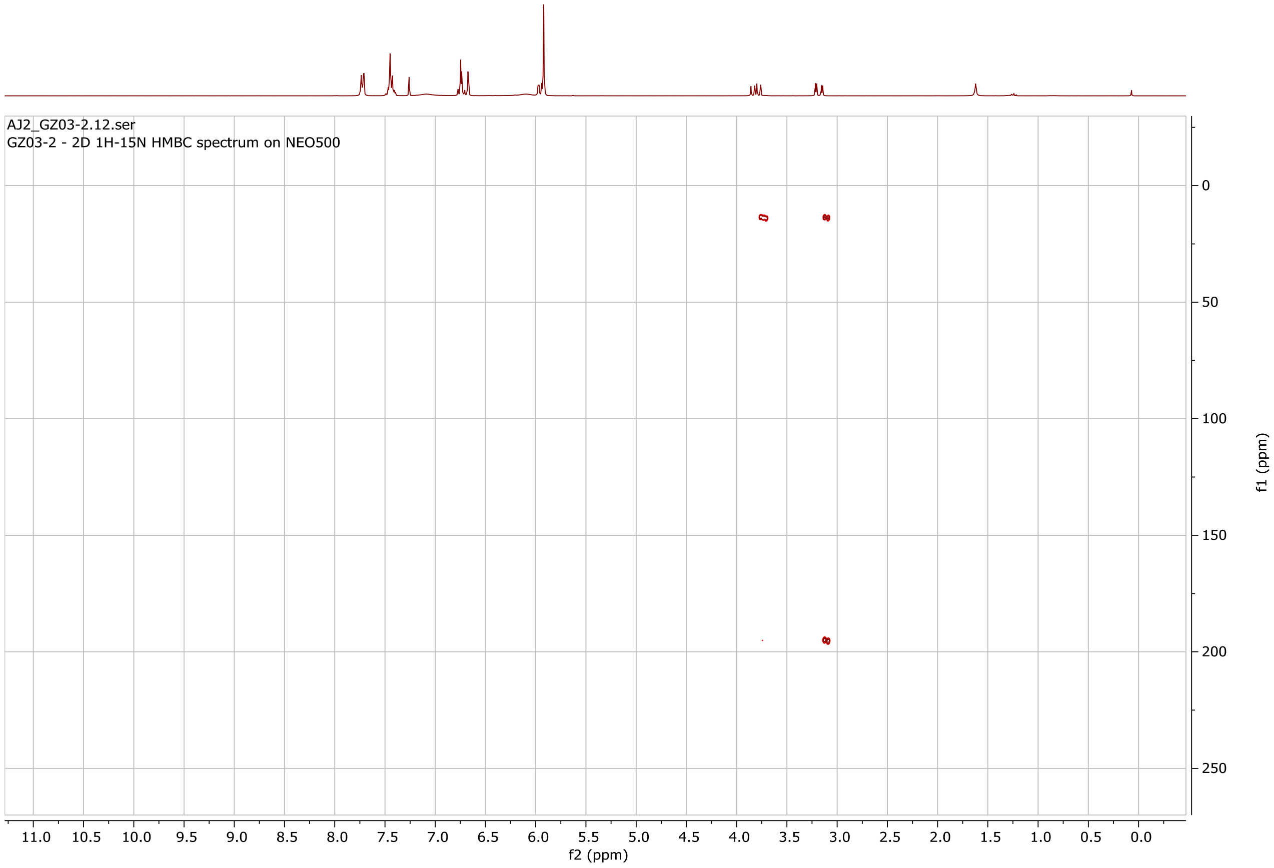

Mass Spectrometry of **4a**

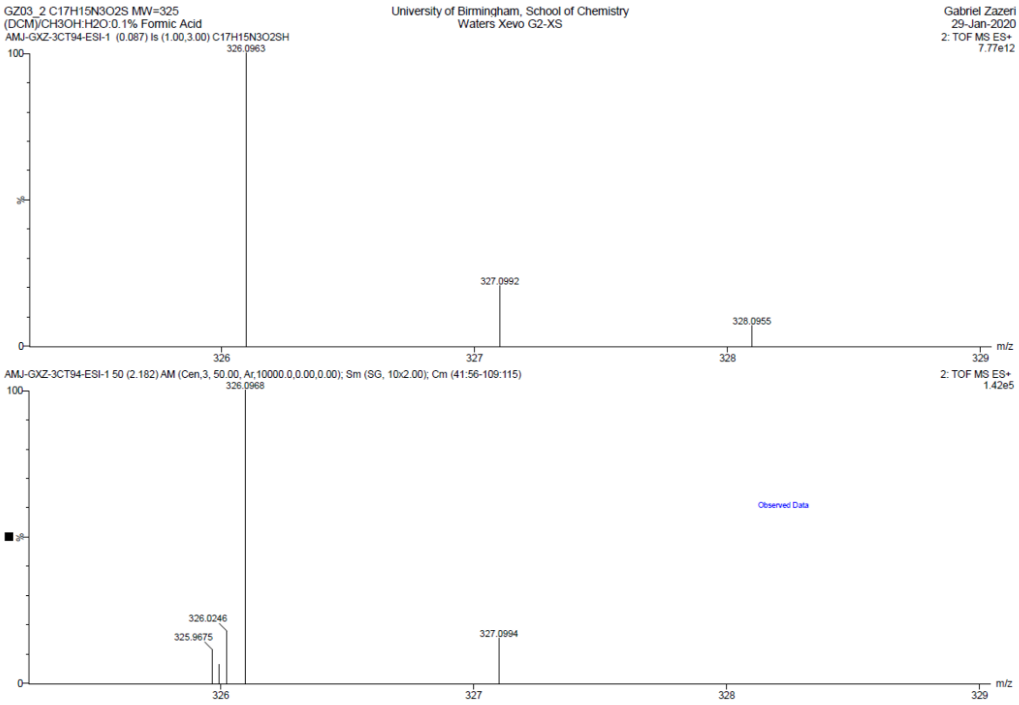

^{1}H NMR spectrum of **4c** (500 MHz, DMSO-d_6)

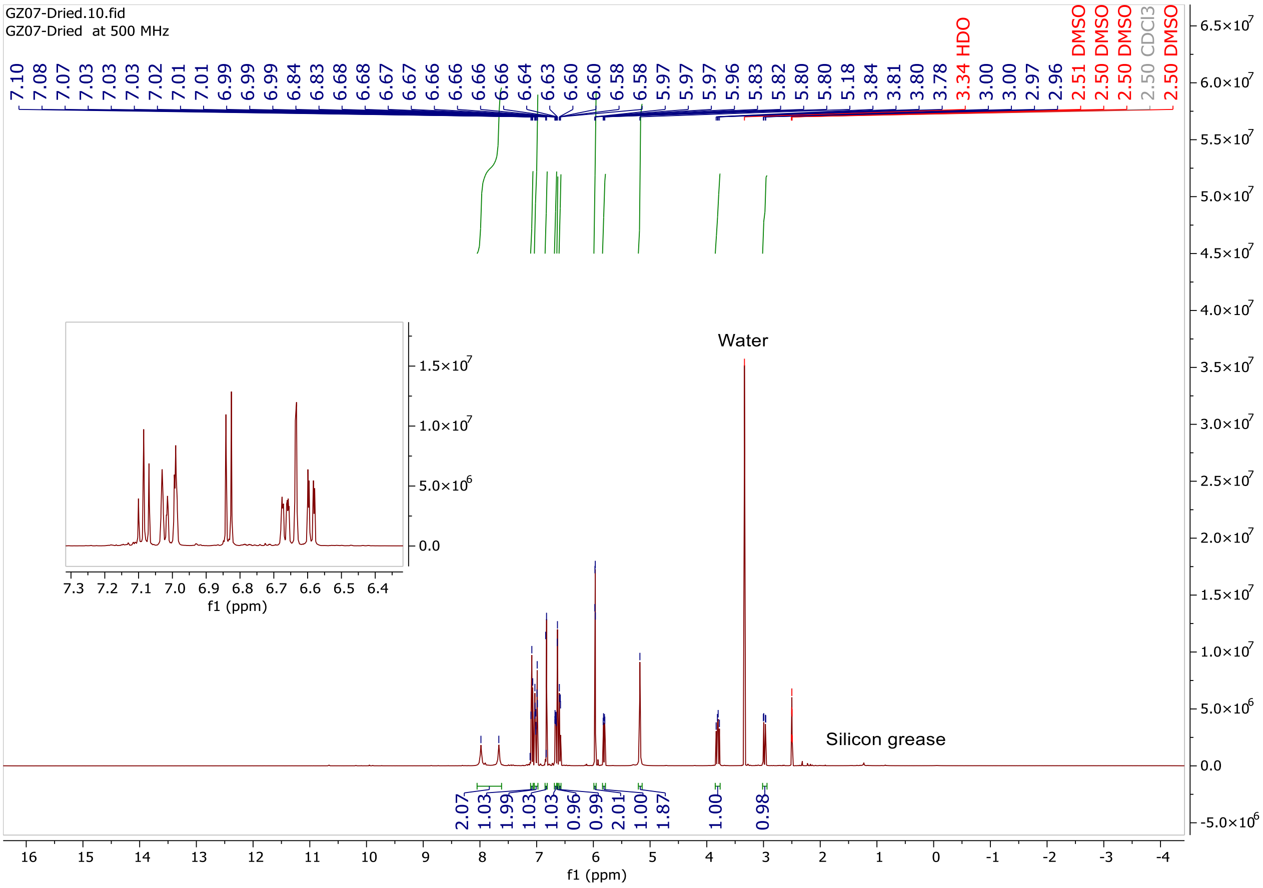

^{13}C NMR spectrum of **4c** (126 MHz, DMSO-d_6)

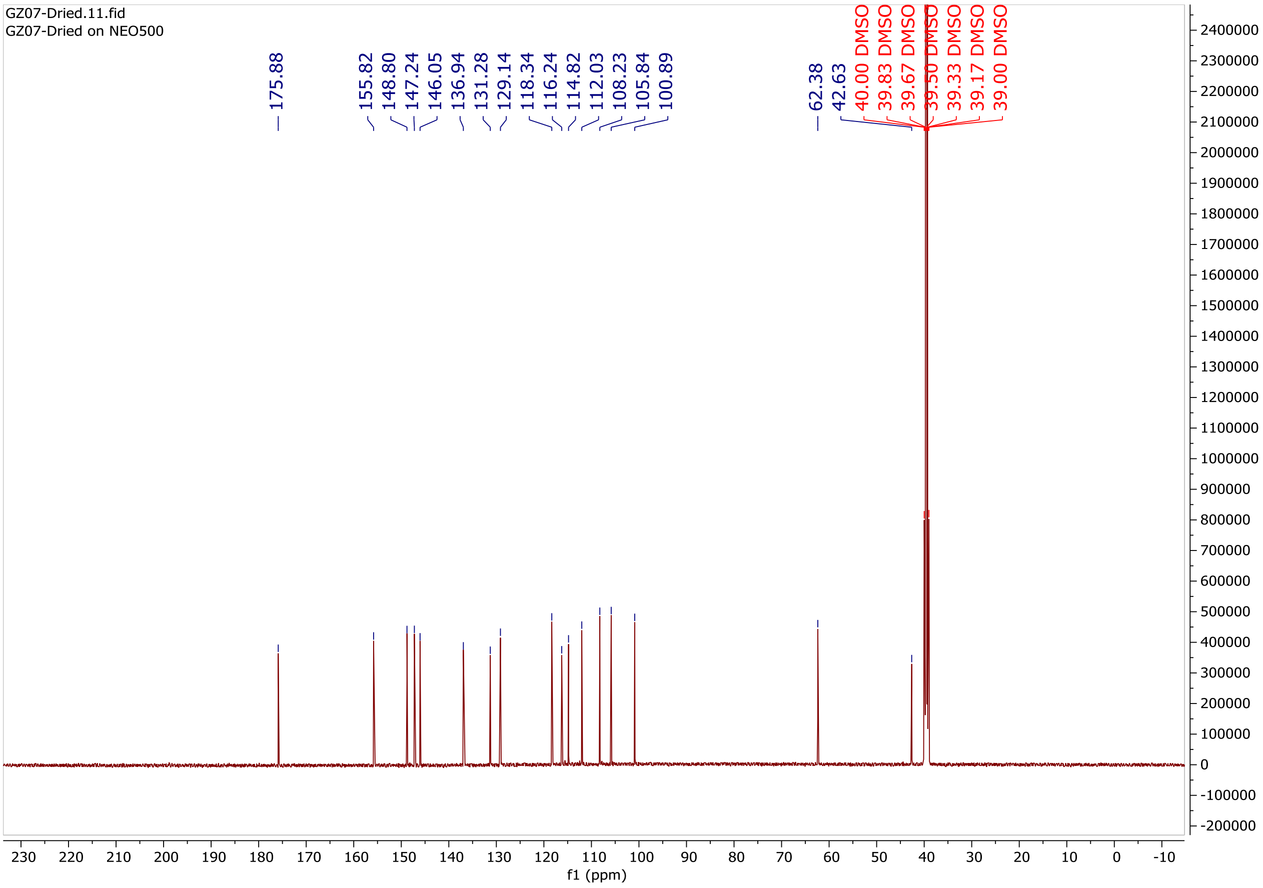

^{1}H-^{1}H COSY NMR spectrum of **4c**

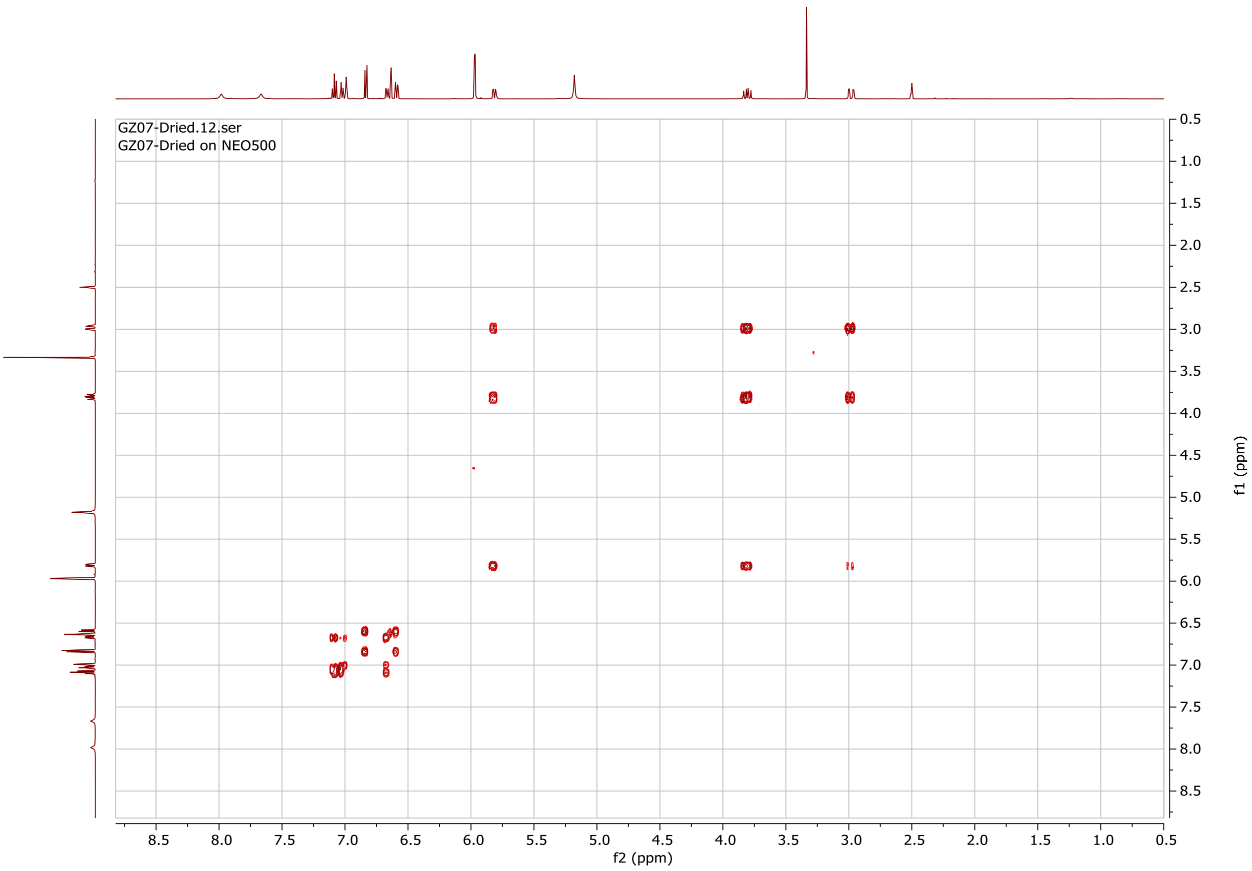

^{1}H-^{13}C HSQC NMR spectrum of **4c**

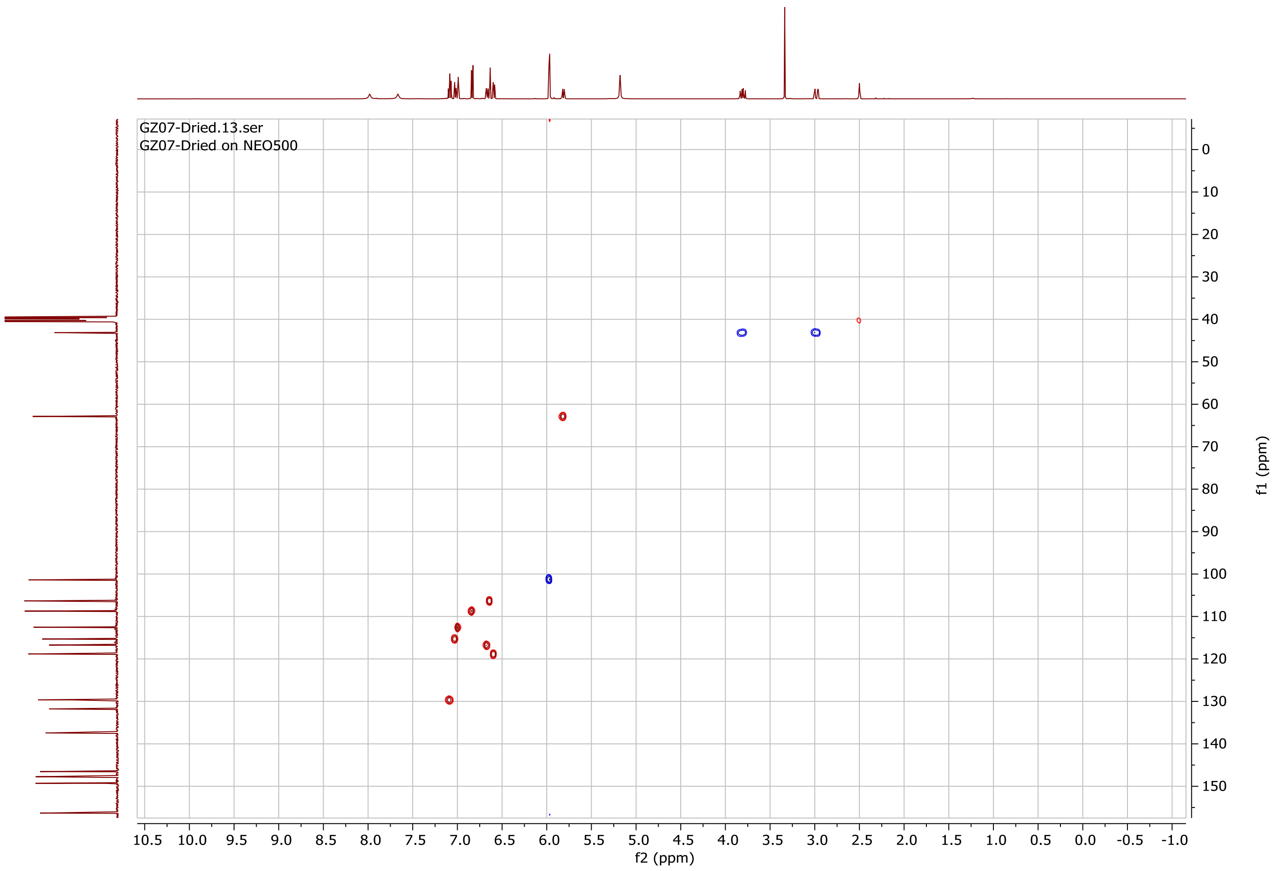

^{1}H-^{13}C HMBC NMR spectrum of **4c**

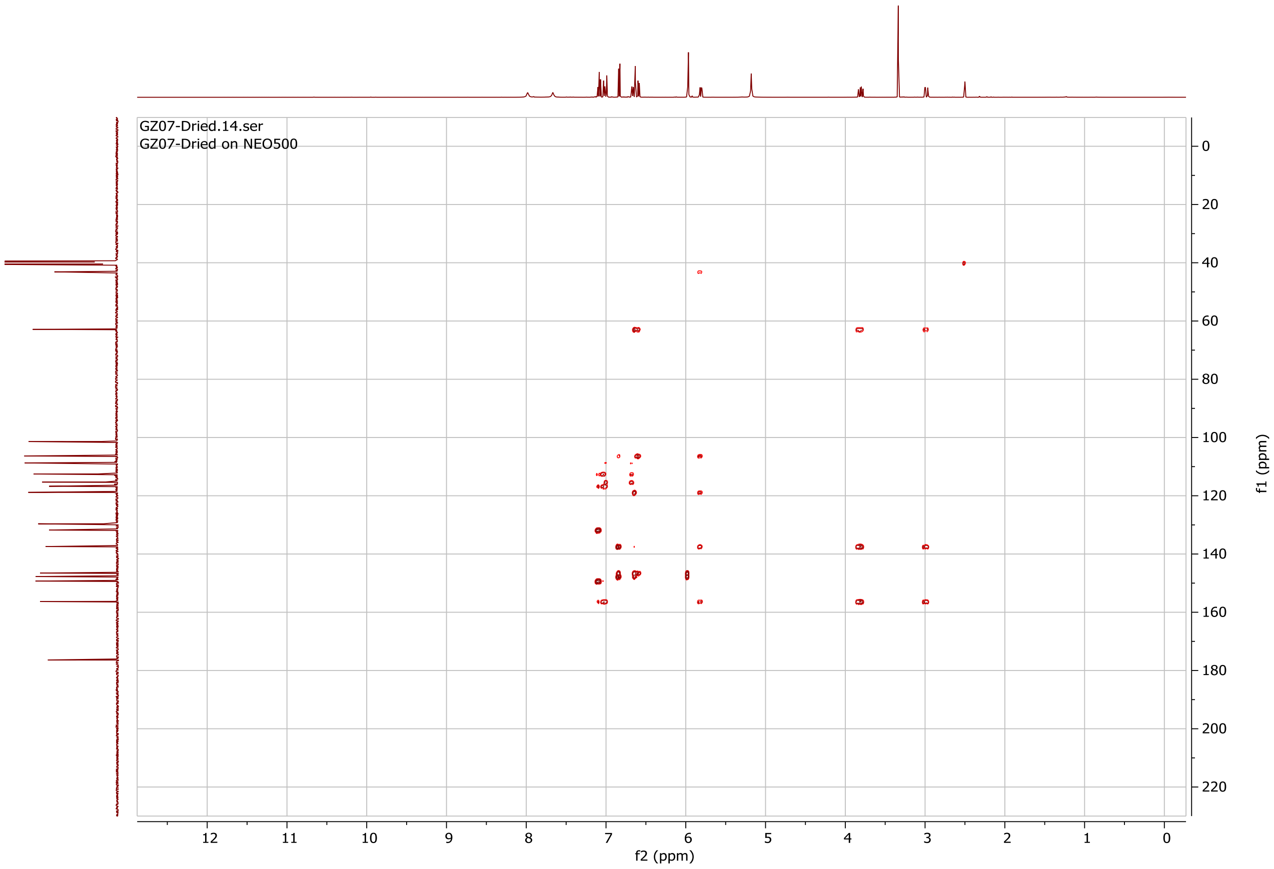

^{1}H-^{15}N HSQC NMR spectrum of **4c**

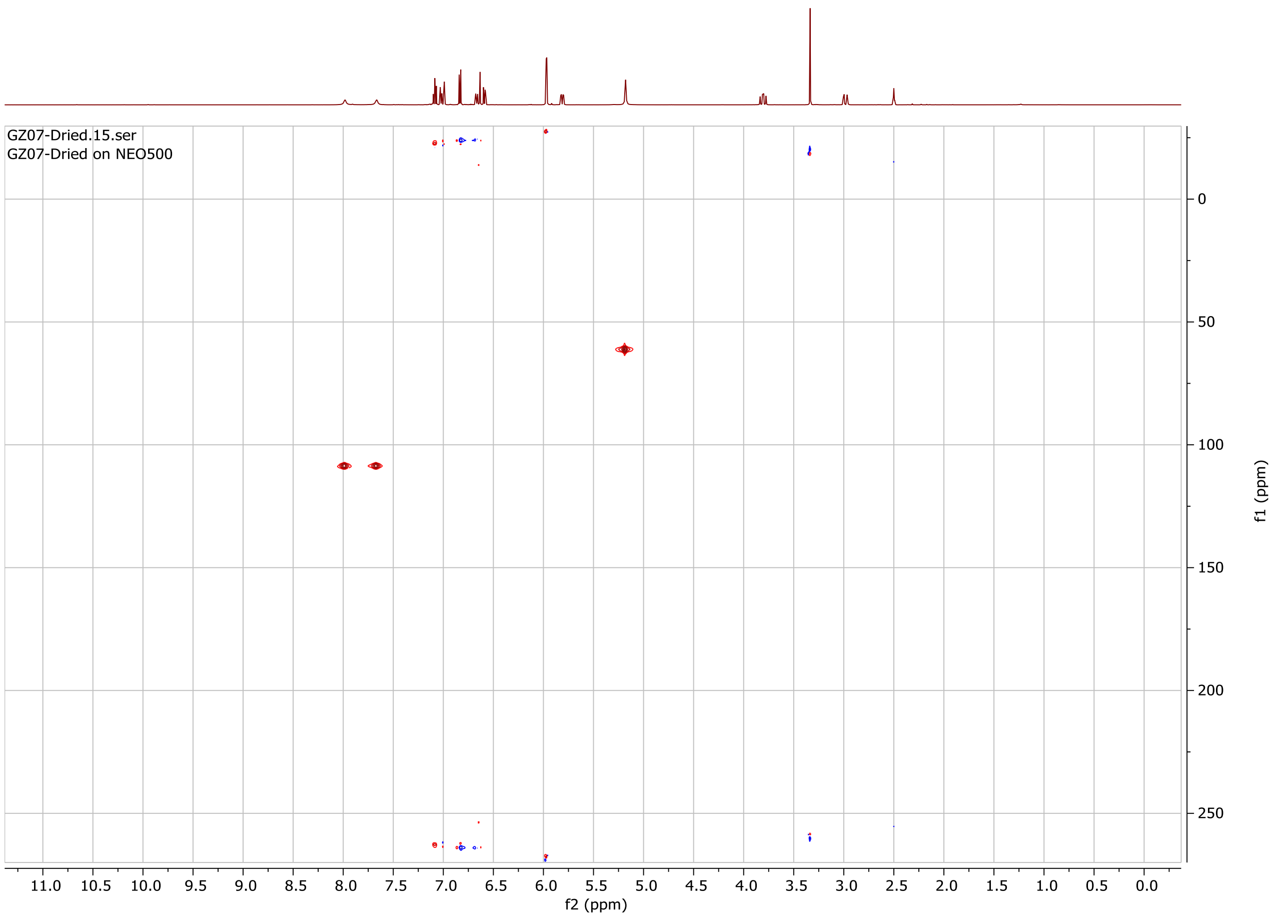

^{1}H-^{15}N HMBC NMR spectrum of **4c**

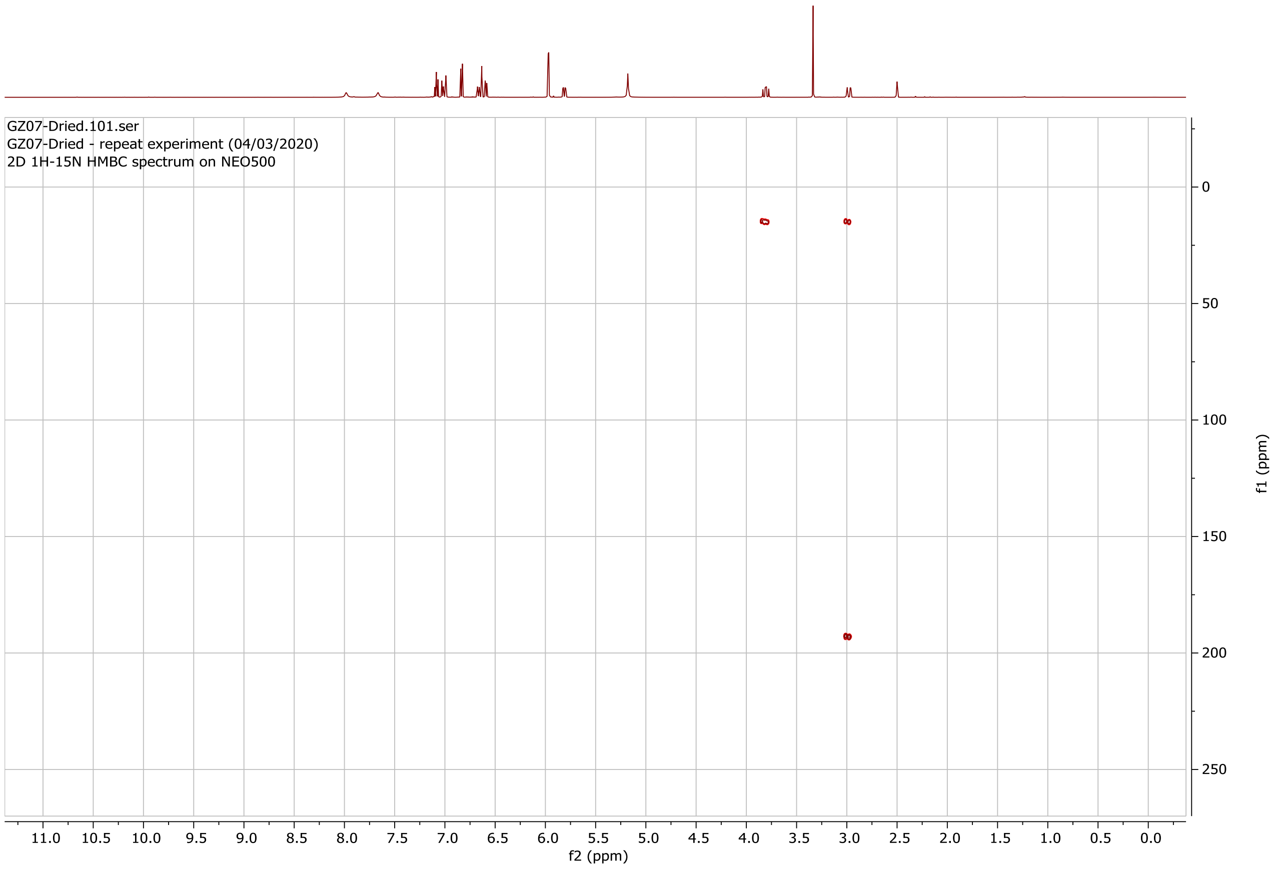

Mass Spectrometry of **4c**

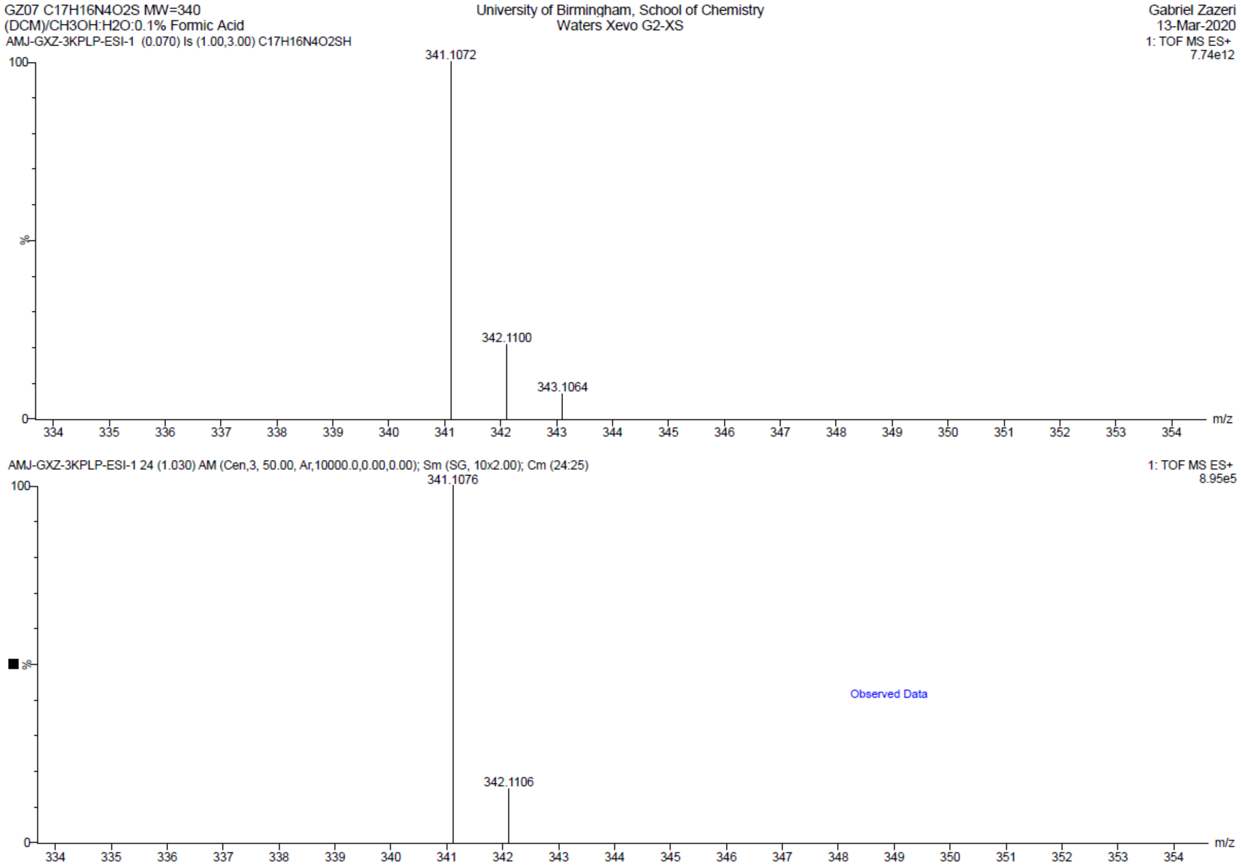

^{1}H NMR spectrum of **7a** (300 MHz, $CDCl_3$)

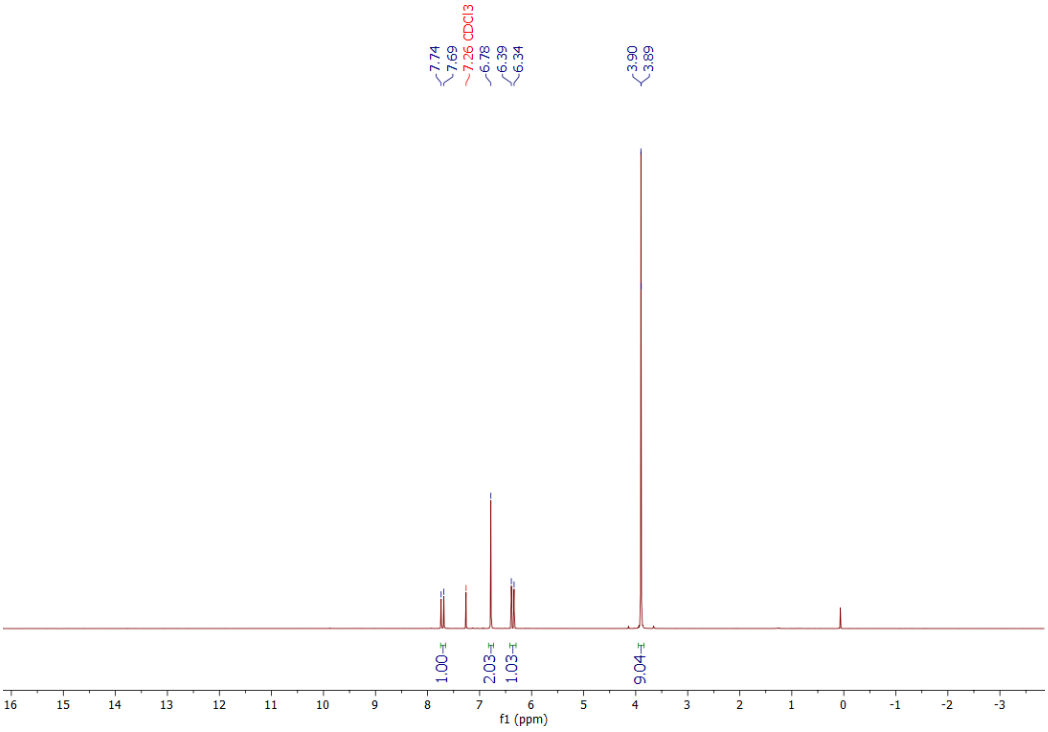

^{13}C NMR spectrum of **7a** (101 MHz, $CDCl_3$)

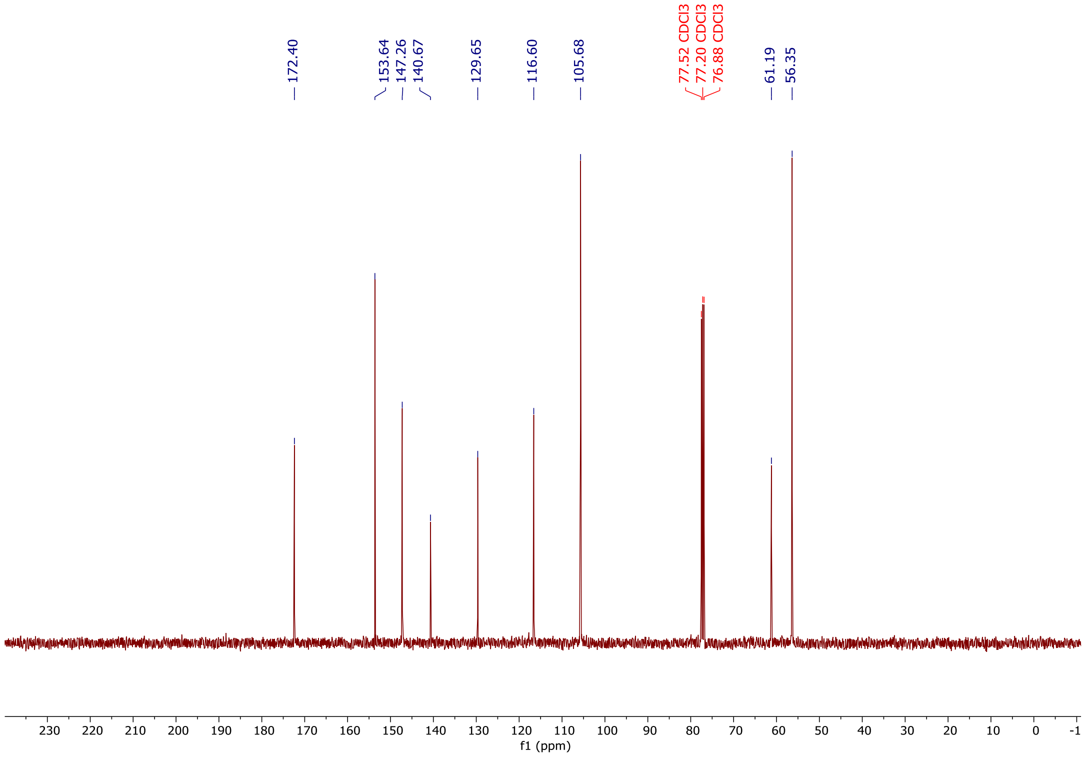

Mass Spectrometry of **7a**

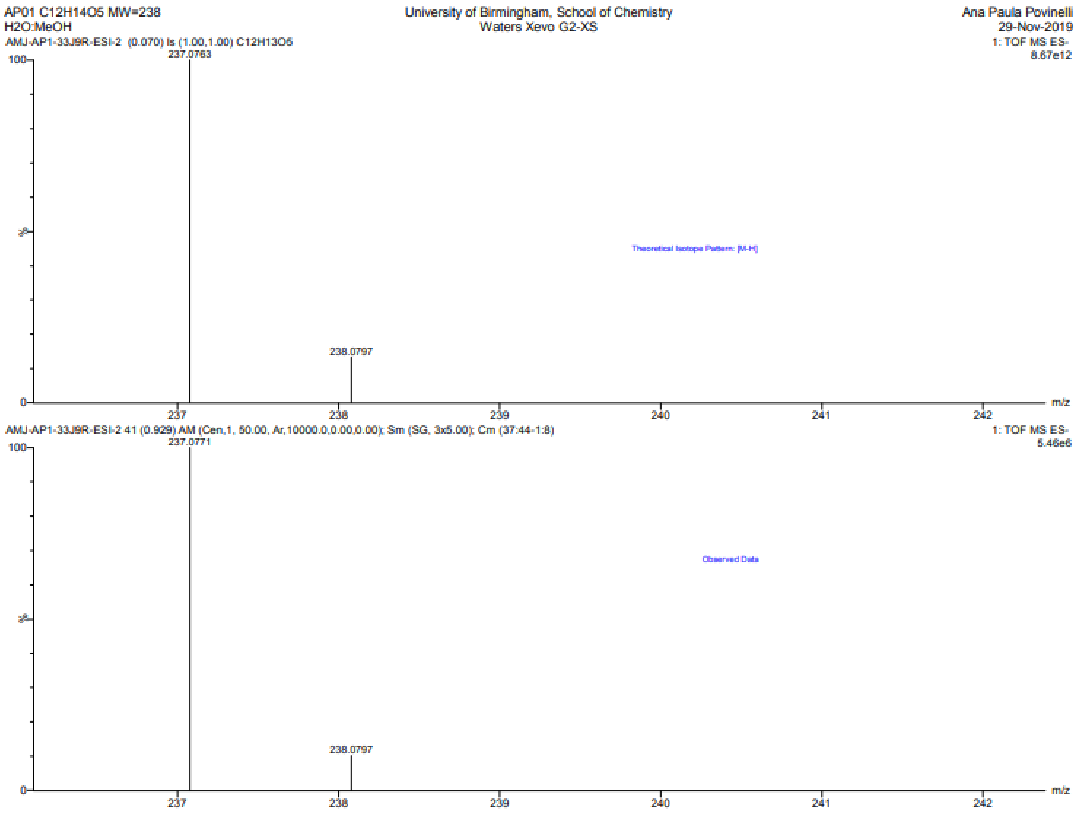

^{1}H NMR spectrum of **7b** (300 MHz, CD_3OD)

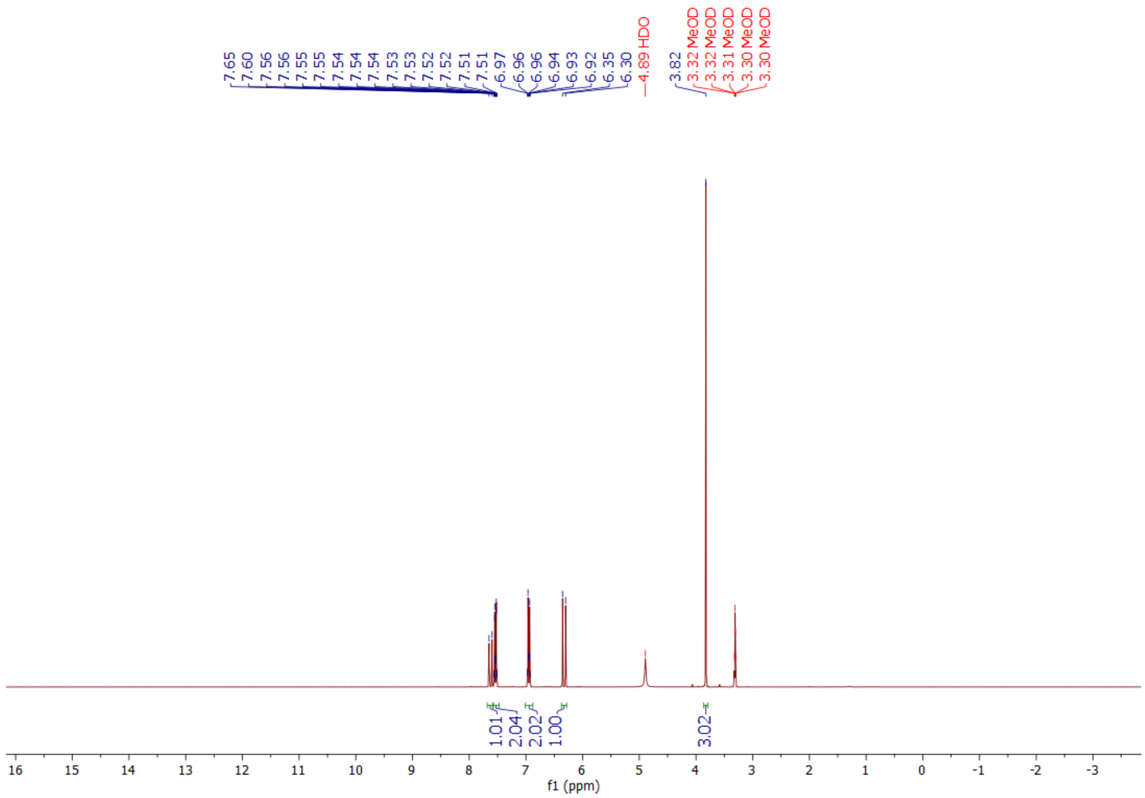

^{1}H NMR spectrum of **8a** (300 MHz, $CDCl_3$)

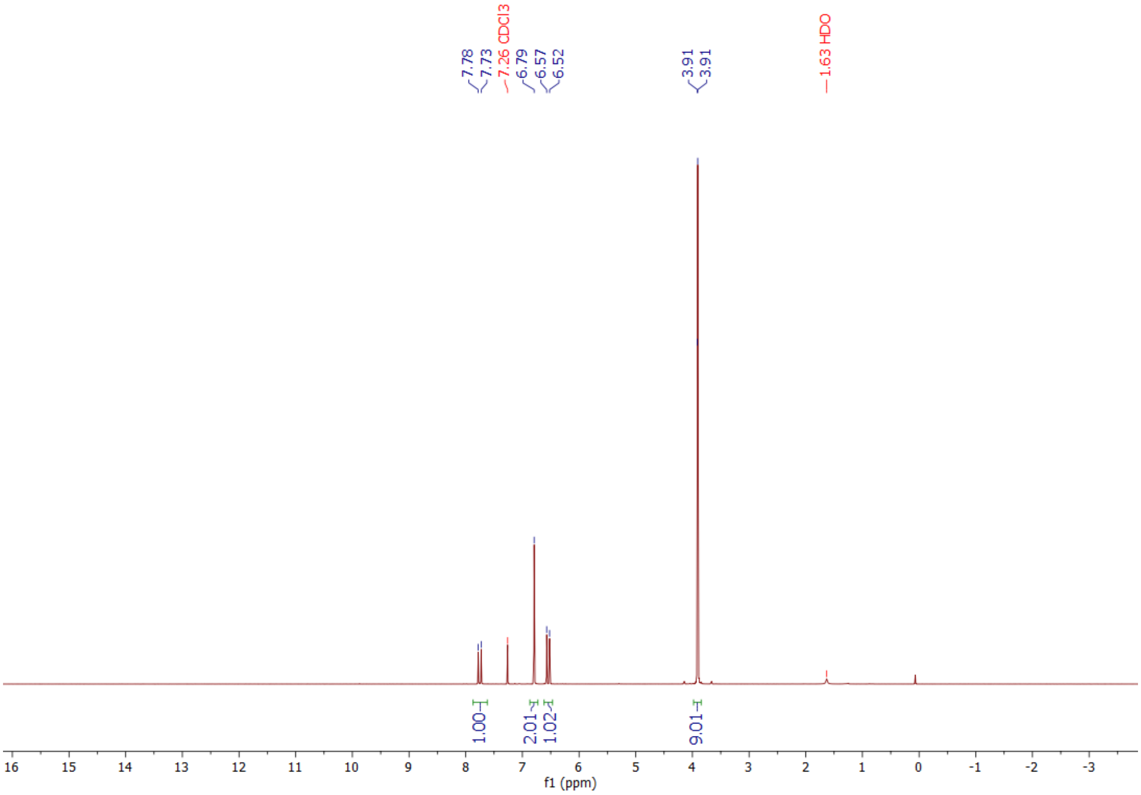

^{1}H NMR spectrum of **8b** (300 MHz, CD_3OD)

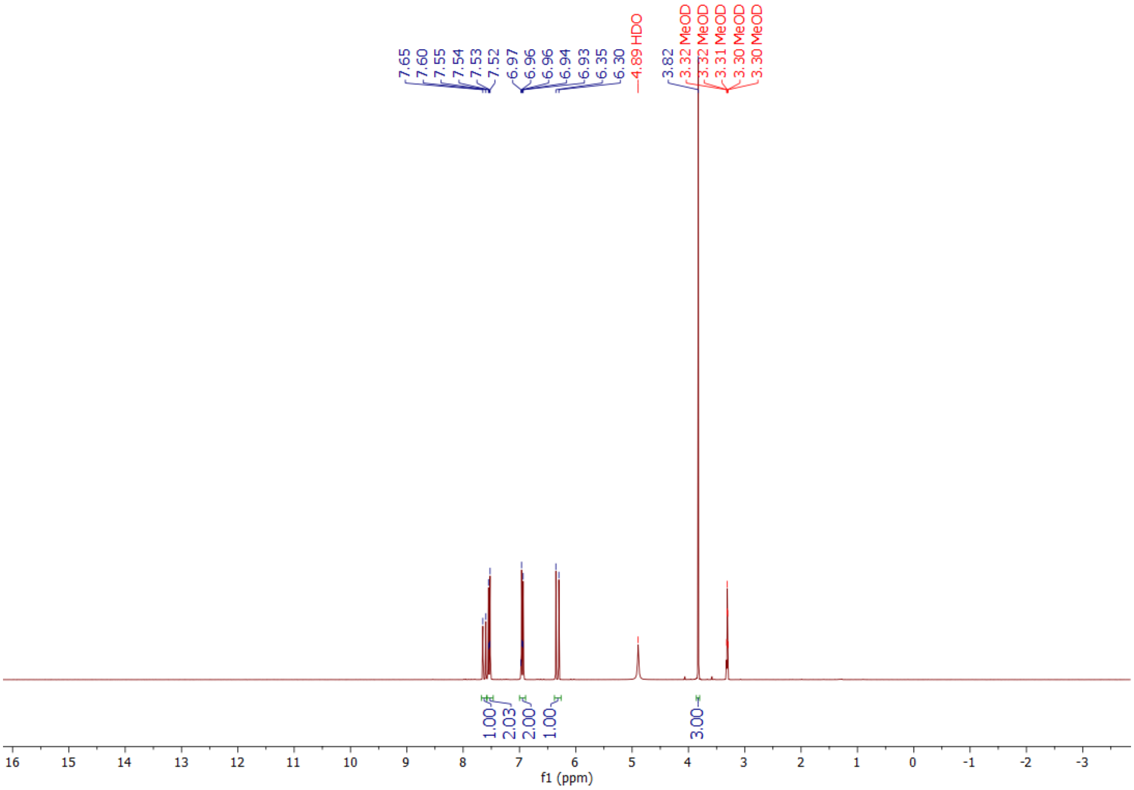

^{1}H NMR spectrum of **9a** (300 MHz, $CDCl_3$)

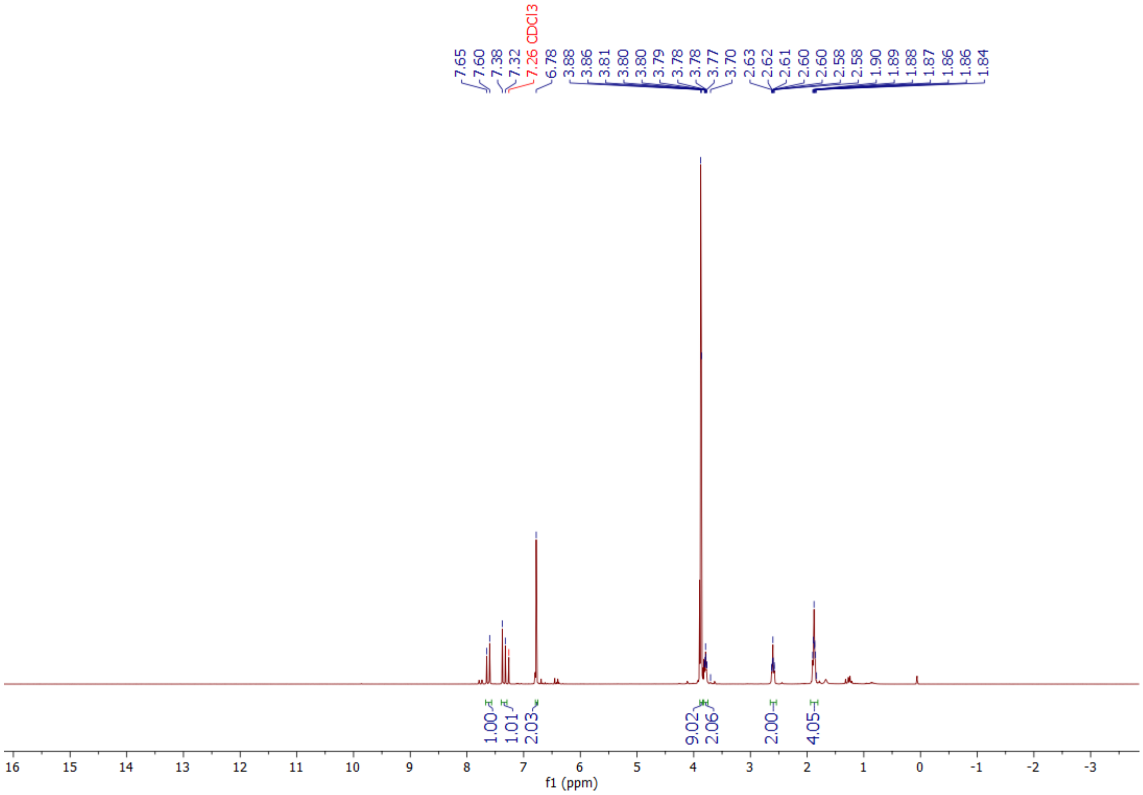

^{1}H NMR spectrum of **9b** (300 MHz, $CDCl_3$)

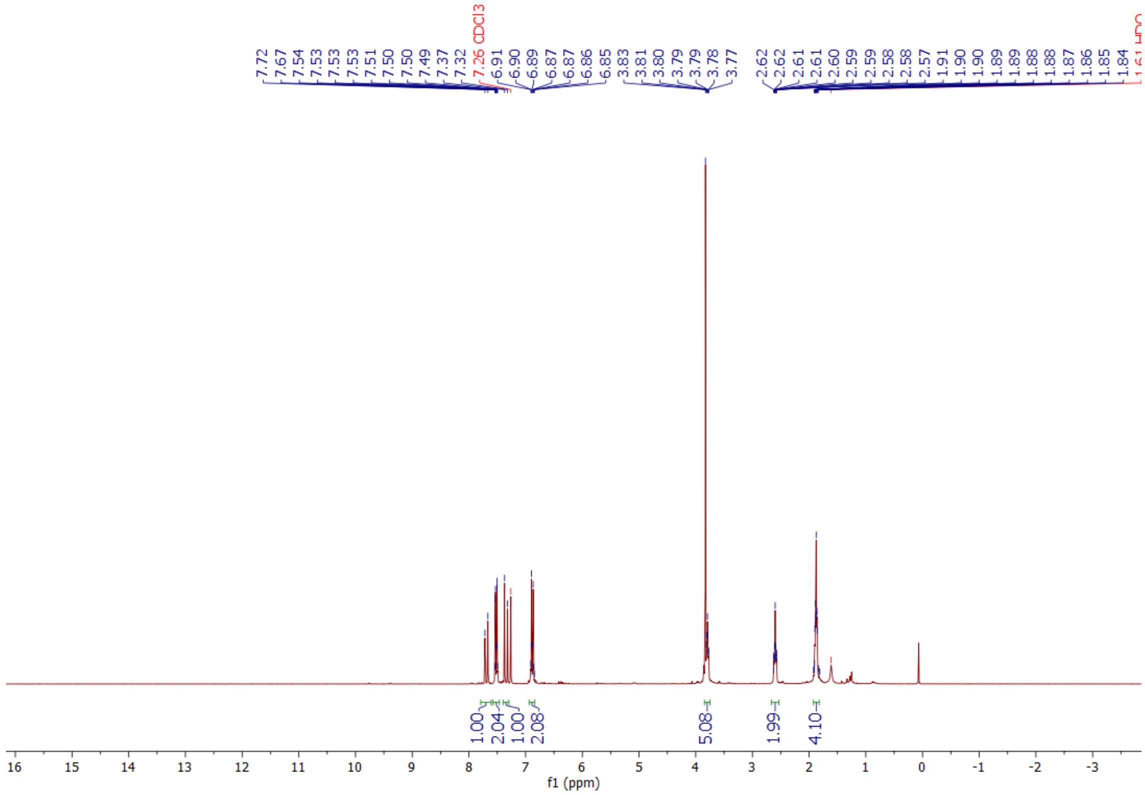

^{1}H NMR spectrum of **10** (300 MHz, $CDCl_3$)

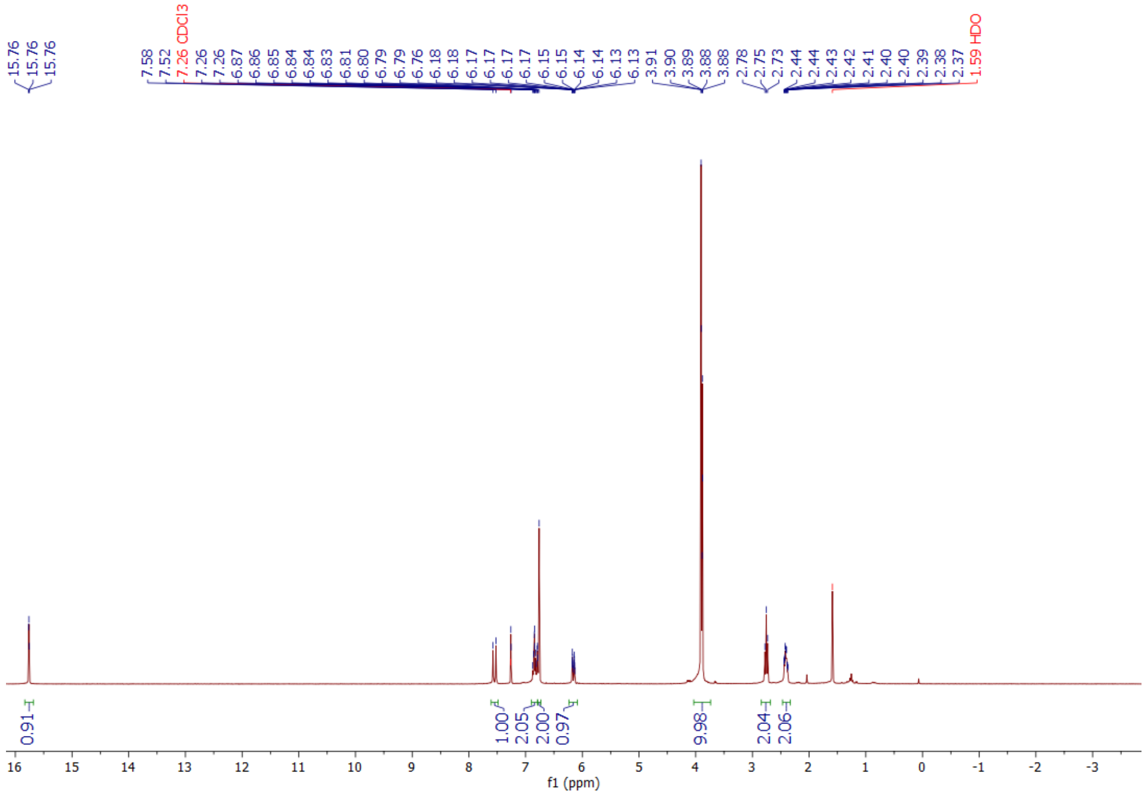

Molecular docking

cation-π interaction between Lys77 of IL-1B and **3a**, **3c** and **9b**.

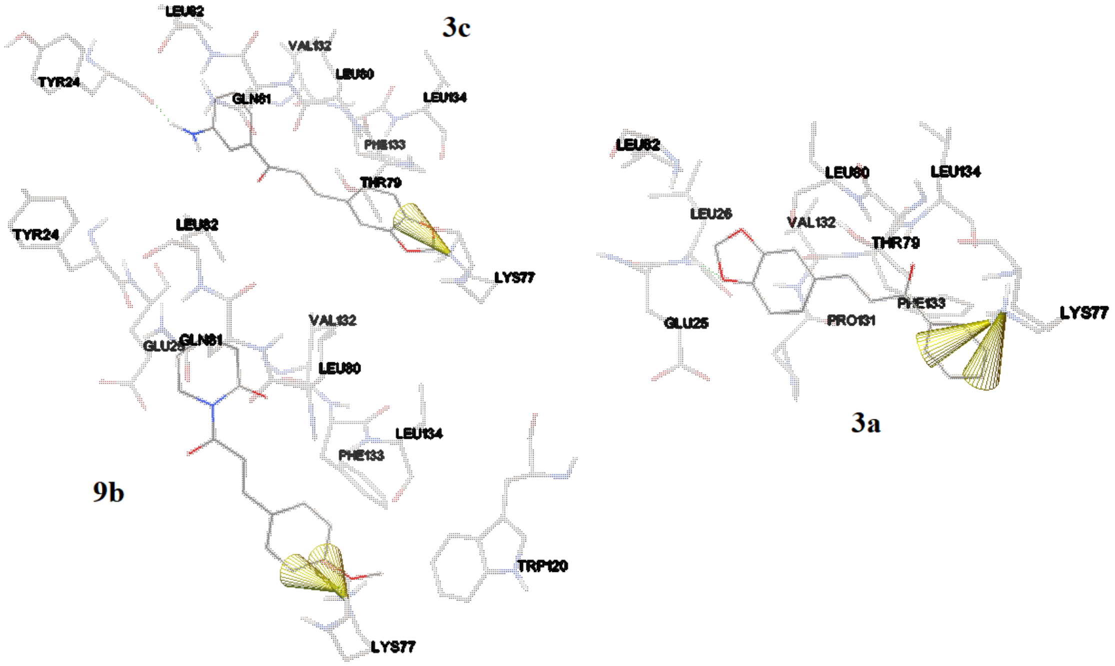

Ab initio Calculation of selected dihedral torsional angles

3a

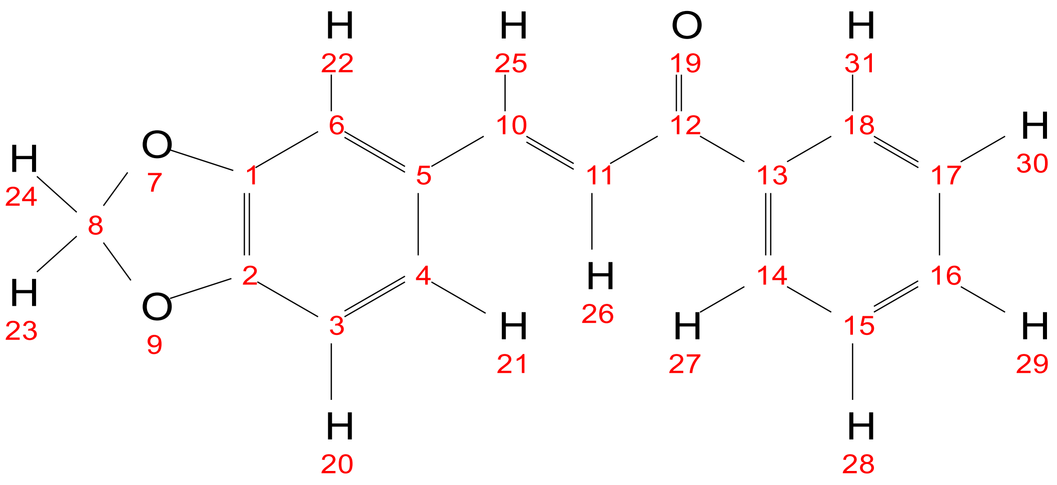

Dihedral	Torsional angle
14-15-16-17	0.228
18-13-12-11	168.513
19-12-11-10	-5.721
10-5-6-1	179.978
7-8-9-2	-8.186
2-3-4-5	0.087

3b

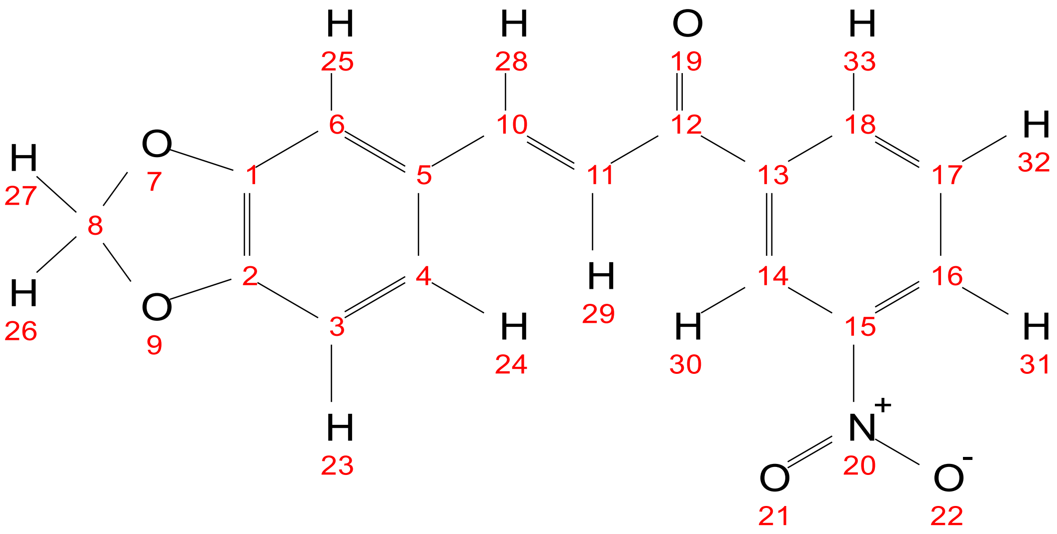

Dihedral	Torsional angle
21-15-22-20	-0.036
22-20-15-16	-2.658
16-17-18-13	0.112
18-13-12-19	-0.218
18-13-12-11	179.987
11-10-5-6	178.969
6-1-7-8	177.766
8-9-2-3	-177.792

3c

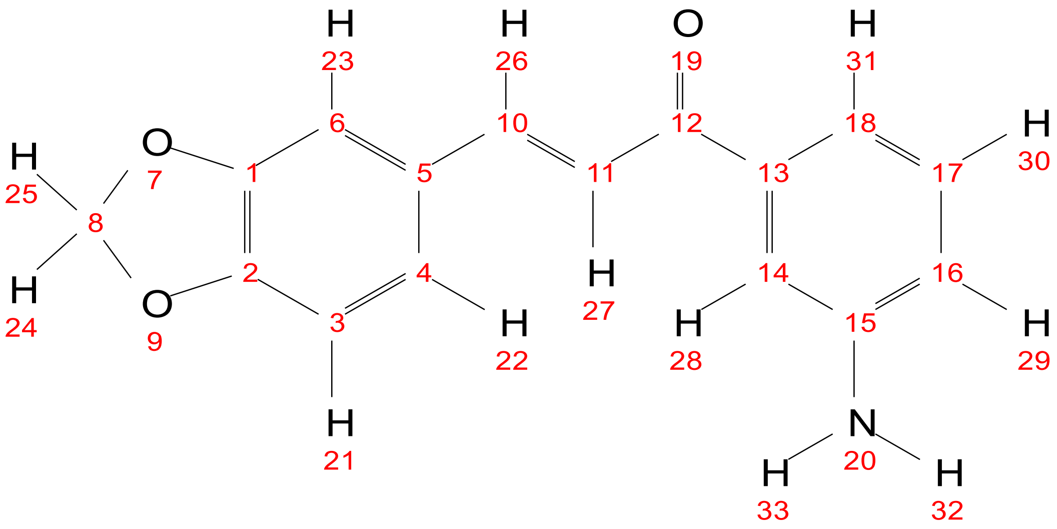

Dihedral	Torsional angle
20-15-16-17	-179.849
16-17-18-13	0.012
18-13-12-19	-0.793
18-13-12-11	179.225
11-10-5-6	179.596
5-6-1-7	-179.991
8-9-2-3	-179.961

4a

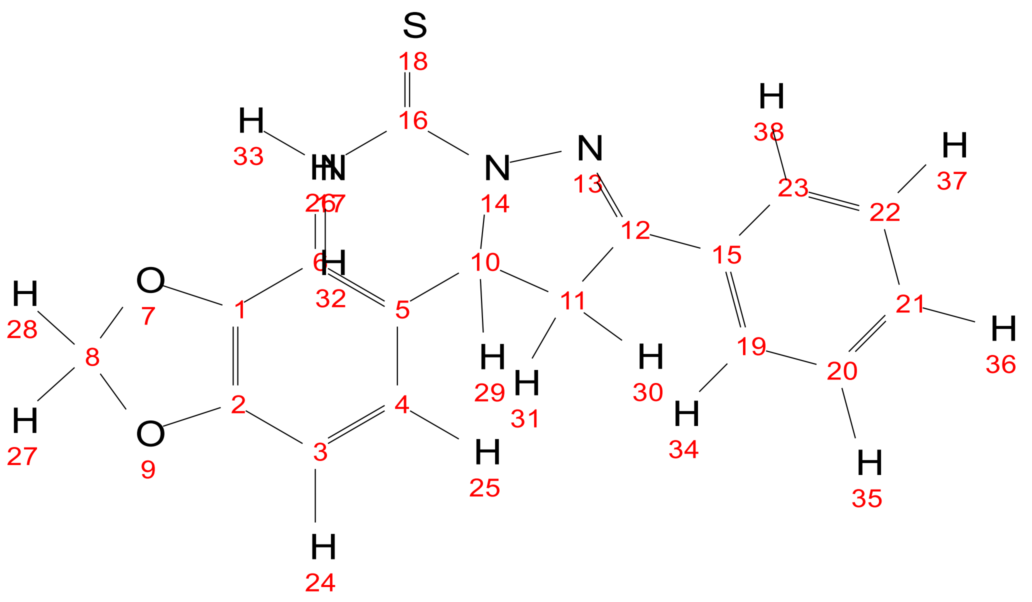

Torsional angle of the dihedral of **4a** simulated in H_2O

Dihedral	Torsional angle
19-20-21-22	-0.067
23-15-12-11	177.810
12-13-14-10	0.626
13-14-10-11	-1.407
14-11-13-10	-0.950
14-17-16-18	-178.286
11-10-5-4	-74.257
11-12-15-19	-2.859
4-3-2-9	176.492
8-7-1-32	-176.820

Torsional angle of the dihedral of 5-membered ring of **4a** simulated in $CDCl_3$

Dihedral	Torsional angle
13-14-10-11	4.838
14-11-13-10	-3.046
12-13-14-10	-2.841

4c

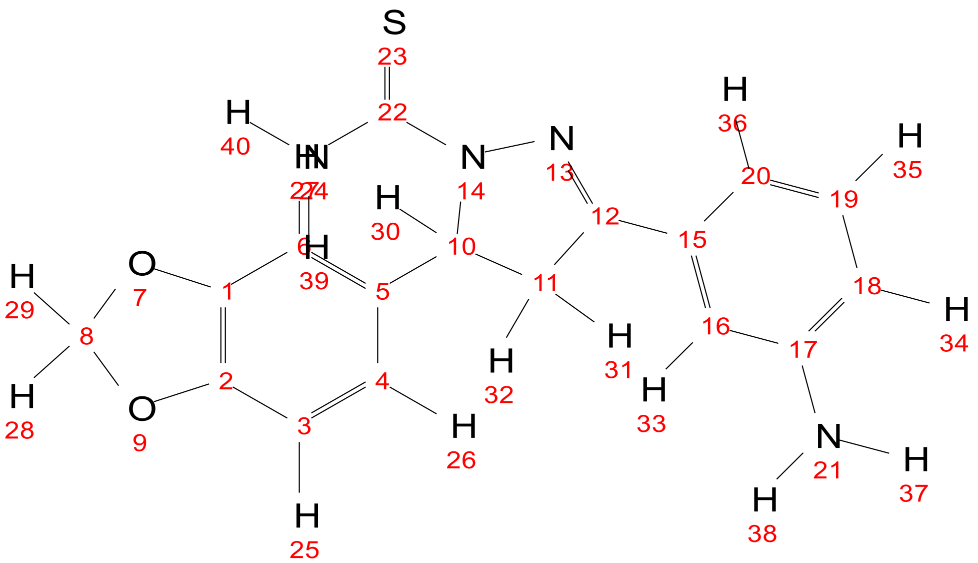

Dihedral	Torsional angle
21-17-18-19	176.732
20-15-12-11	-172.194
12-13-14-10	0.626
13-10-14-11	-1.407
13-14-10-11	102.997
6-1-7-8	173.361
8-9-2-3	-178.833
6-5-4-3	0.268

9a

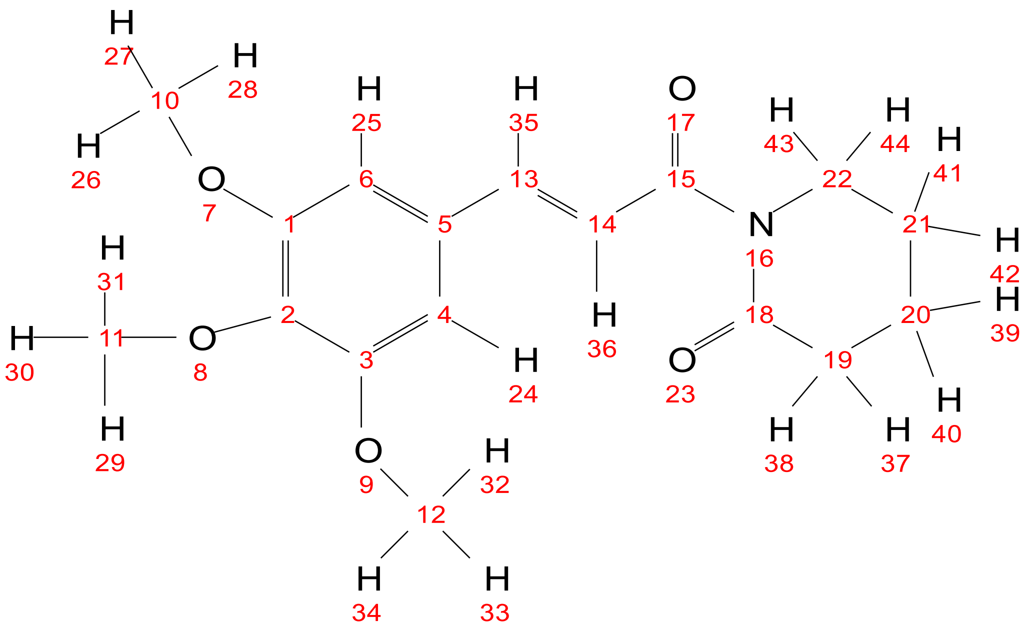

Dihedral	Torsional angle
20-21-22-16	-48.558
22- 16-15-17	9.655
16-15-14-13	-173.008
13-5-6-1	179.762
10-7-1-2	73.431
11-8-2-3	-119.435
9- 12-3-4	-134.562
15-16-22-21	-167.467

9b

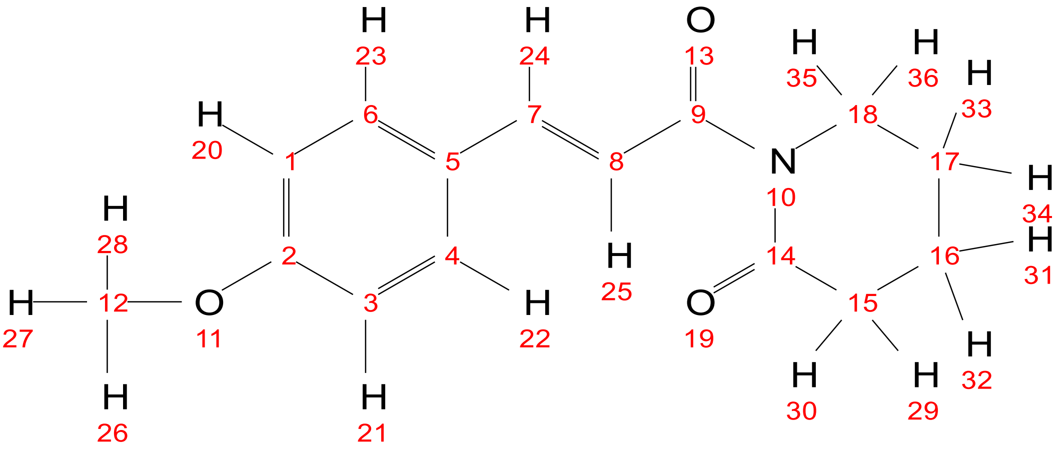

Dihedral	Torsional angle
19-14-15-16	-173.441
17-18-10-9	-168.002
13-9-8-7	12.815
5-5-1-2	0.191
12-11-2-3	-179.427
8-7-5-6	179.783
19-14-15-16	-173.441
17-18-10-9	-168.002

10

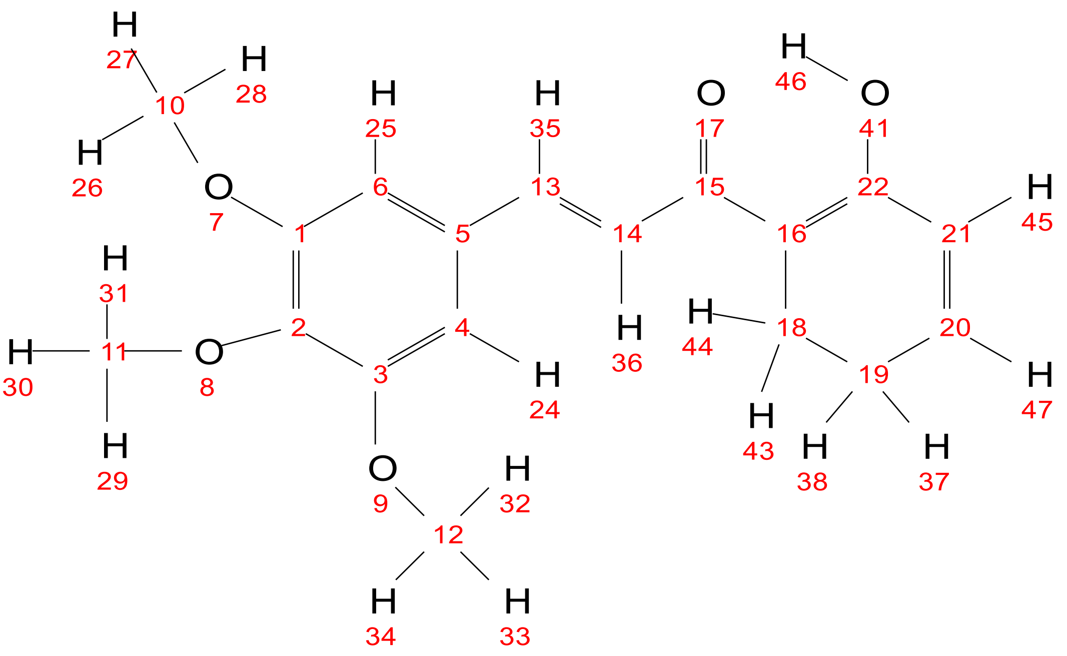

Dihedral	Torsional angle
18-19-20-21	28.591
41-22-16-15	1.471
17-15-14-13	-5.019
15-14-13-5	179.702
10-7-1-2	72.840
11-8-2-3	-106.728
12-9-3-4	112.209
14-15-16-22	176.508

References

1. Williams, D.B.G.; Lawton, M. Drying of Organic Solvents: Quantitative Evaluation of the Efficiency of Several Desiccants. *J. Org. Chem.* **2010**, *75*, 8351-8354.
2. Sun, L.-D.; Wang, F.; Dai, F.; Wang, Y.-H.; Lin, D.; Zhou, B. Development and mechanism investigation of new piperlongumine derivative as a potent anti-inflammatory agent. *Biochem. Pharmacol.* **2015**, *95*, 156-169.
3. Mathew, A.; Sheeja T. L., M.; Kumar T., A.; Radha, K. Design, Synthesis and Biological Evaluation of Pyrazole analogues of Natural Piperine. *Hygeia J. D. Med.* **2011**, *3*, 48-56.
